# Retinal organoids with X-linked retinoschisis *RS1* (E72K) mutation exhibit a photoreceptor developmental delay and are rescued by gene augmentation therapy

**DOI:** 10.1186/s13287-024-03767-4

**Published:** 2024-05-31

**Authors:** Chunwen Duan, Chengcheng Ding, Xihao Sun, Shengru Mao, Yuqin Liang, Xinyu Liu, Xiaoyan Ding, Jiansu Chen, Shibo Tang

**Affiliations:** 1 Aier School of Ophthalmology, Central South University, Changsha, Hunan China; 2 Aier Eye Institute, Changsha, Hunan China; 3 The Second Xiangya Hospital of Central South University, Changsha, Hunan China; 4 State Key Laboratory of Ophthalmology, Zhongshan Ophthalmic Center, Sun Yat-Sen University, Guangzhou, China; 5 Key Laboratory for Regenerative Medicine, Ministry of Education, Jinan University, Guangzhou, Guangdong China; 6 Guangzhou Aier Eye Hospital, Guangzhou, Guangdong China

**Keywords:** X-linked retinoschisis (XLRS), Retinal organoids (ROs), Human induced pluripotent stem cells (hiPSCs), Photoreceptor, Gene augmentation therapy

## Abstract

**Background:**

X-linked juvenile retinoschisis (XLRS) is an inherited disease caused by *RS1* gene mutation, which leads to retinal splitting and visual impairment. The mechanism of *RS1*-associated retinal degeneration is not fully understood. Besides, animal models of XLRS have limitations in the study of XLRS. Here, we used human induced pluripotent stem cell (hiPSC)-derived retinal organoids (ROs) to investigate the disease mechanisms and potential treatments for XLRS.

**Methods:**

hiPSCs reprogrammed from peripheral blood mononuclear cells of two *RS1* mutant (E72K) XLRS patients were differentiated into ROs. Subsequently, we explored whether *RS1* mutation could affect RO development and explore the effectiveness of *RS1* gene augmentation therapy.

**Results:**

ROs derived from *RS1* (E72K) mutation hiPSCs exhibited a developmental delay in the photoreceptor, retinoschisin (RS1) deficiency, and altered spontaneous activity compared with control ROs. Furthermore, the delays in development were associated with decreased expression of rod-specific precursor markers (NRL) and photoreceptor-specific markers (RCVRN). Adeno-associated virus (AAV)-mediated gene augmentation with *RS1* at the photoreceptor immature stage rescued the rod photoreceptor developmental delay in ROs with the RS1 (E72K) mutation.

**Conclusions:**

The RS1 (E72K) mutation results in the photoreceptor development delay in ROs and can be partially rescued by the *RS1* gene augmentation therapy.

**Supplementary Information:**

The online version contains supplementary material available at 10.1186/s13287-024-03767-4.

## Introduction

X-linked retinoschisis (XLRS) is an X-linked inherited vitreoretinal disease with an estimated prevalence ranging from 1:5,000 to 1:25,000 worldwide [1, 2]. Its characteristic features include central vision loss, fovea-macular cystic schisis cavities, splitting of neural retinal layer, and decreased b-wave amplitude of electroretinogram (ERG) [3–5]. XLRS is prevalent among males with school-age reading difficulties [1]. Females carrying XLRS heterozygous are considered asymptomatic [1, 6]. The best corrected visual acuities (BCVA) of affected men vary from 20/20 to 20/600, and progressive visual acuities (VA) loss range from 0.22 to 0.5 letters per year [7, 8]. Some patients develop serious complications, such as retinal detachment, vitreous hemorrhage, and retinal tears, with the progression of the disease, which can lead to dramatic vision loss [5]. XLRS patients suffer from long-term vision problems. While improving VA or slowing vision loss remains a primary goal of current research, effective clinical treatments for XLRS are still under development.

A total of 452 different mutations were found in the *RS1* gene, including point mutations (nonsense or missense), deletions, insertions, or splice site alterations (https://databases.lovd.nl/shared/genes/RS1). Retinoschisin (RS1) is a secreted protein that can form an octamer, and its functions are related to cell adhesion and maintenance of retinal structure integrity [9, 10]. However, the molecular mechanism of RS1 remains elusive. Different forms of mutation in the *RS1* gene lead to the diverse structures of the RS1 protein. Studies have shown that some mutant RS1 proteins exhibit functional abnormalities. These abnormalities can manifest in two ways: Impaired secretion: Certain mutations prevent the RS1 protein from being properly released from the cells where it is produced. Defective octamer formation: Other mutations hinder the RS1 protein’s ability to form functional octameric structures, which are essential for its role in the retina [11]. This indicates differences in functionality among mutant RS1 proteins. The mutation c.214G > A (p.E72K) of *RS1* is the frequently reported hotspot mutation [4, 10, 12, 13]. Therefore, it is important to investigate c.214G > A to expand our understanding of the mechanism of XLRS.

Currently, more than seven mouse models have been established, including *Rs1* gene knock-out (*Rs1*-KO), *Rs1* gene knock-in (*Rs1*-KI), and *Rs1* nucleotide or exon deletions [5]. Despite effectively modeling key aspects of XLRS, including RS1 deficiency and characteristic retinal abnormalities, these animal models have limitations. The relatively low median amino acid sequence identity of 78.5% between humans and mice can lead to phenotypic discrepancies under certain conditions, highlighting the need for alternative models that more closely resemble the human disease [14]. 

Mouse and human retinas are not equivalent since the mouse is a nocturnal species with a rare cone distributed over the whole retina and an absence of a cone-rich macula [15]. In addition, some mouse models with human mutant orthologs failed to display disease-relevant phenotypes as humans [16]. However, previous studies have shown that XLRS might have some problems with photoreceptors [17, 18]. Moreover, retinal signs of XLRS have been described in infants in some cases [13, 19], suggesting that lesions developed even before birth. Thus, the mouse model may not be the best choice for studying XLRS.

Since Haas first described XLRS in 1898, research on its mechanism and treatment has been ongoing [1]. Various experimental treatments have been explored, including carbonic anhydrase inhibitors (CAIs) and *RS1* gene augmentation. Abnormal schisis or splitting in XLRS responds to CAIs, which are thought to function through retinal pigment epithelium (RPE) [2, 20]. However, there is little or no improvement in VA in CAI-treated XLRS patients [21–23]. XLRS is a genetic disorder caused by mutations in a single gene, *RS1*, located on the distal short arm of the X chromosome at Xp22.1. This gene encodes the protein retinoschisin, which plays a critical role in retinal development and function [1]. Therefore, the idea of gene augmentation therapy arises spontaneously. Preclinical investigations have indicated that enhancing the normal *RS1* gene can diminish retinal schisis cavities, restore retinal architecture, and enhance retinal function. Furthermore, studies have identified specific types of AAV capable of penetrating the ILM barrier, thereby delivering the therapeutic cargo directly to photoreceptor cells. Data indicates that the AAV-RS1 vector can sustain the expression of RS1 for at least nine months [24–26]. Clinical trials were initiated based on this biological plausibility. A phase I/IIa clinical trial administered the AAV8-RS1 gene therapy to nine XLRS patients aged 23–71 years (ClinicalTrial.gov: NCT02317887). However, no improvement in VA was observed in patients even after 18 months of intravitreal injection [27]. A recent multicenter clinical trial sponsored by Applied Genetics Technology Corporation (AGTC) evaluated the safety and efficacy of rAAV2tYF-CB-hRS1 gene therapy in patients with X-linked retinoschisis (XLRS). The trial enrolled 30 participants (5 children and 25 adults) between the ages of 10 and 79 years (ClinicalTrials.gov identifier: NCT02416622). Similarly, no improvement was found in BCVA, visual fields, or ERG in the eye that received intravitreal injection after a one-year follow-up [28]. Although RS1 is primarily located in the inner segments of the photoreceptor and bipolar cells in the mature retina, it is initially expressed by all retinal neurons except horizontal cells [29–31]. Given the evidence that almost all retinal neurons initially express RS1 and the characterization of earlier onset in patients with XLRS, it was inferred that RS1 may play a role in early retinal development. This could be one of the reasons for the failed improvement of VA in clinical trials. Based on this biological foundation, we sought to administer the *RS1* gene augmentation therapy earlier.

Human-induced pluripotent stem cells (hiPSCs) can differentiate into various cell subtypes. In recent years, due to the development of regenerative medicine, hiPSC-derived organoids, which reproduce the structural and functional characteristics of natural organs, have been developed. The hiPSC-derived retinal organoids (ROs) contain retina-specific cell types and have a laminar structure mimicking the human retina [32]. ROs have shown great potential for basic scientific and medical applications and have been used in several inherited retinopathy studies [33, 34]. Moreover, ROs provide a platform for human retinal development studies, which are limited to scarce human fetal tissue [35]. Furthermore, hiPSC reprogrammed from XLRS patient tissues have an identical genetic background to those of XLRS patients.

In this study, we generated hiPSCs from peripheral blood mononuclear cells (PBMCs) of two *RS1* mutant (c.214G > A) XLRS patients. Further, hiPSCs were differentiated into ROs as a disease model of XLRS. Next, ROs were explored to determine whether the *RS1* mutation affected RO development. Subsequently, ROs were treated with the *RS1* gene augmentation therapy. The resulting ROs demonstrated that XLRS patient-specific iPSC-derived ROs could be a suitable XLRS disease model, which mimicked human retinas with RS1 deficiency. RNA-seq analysis of patient-derived ROs revealed downregulation of genes associated with synapsis, nervous system development, and Class A/1 (Rhodopsin-like) receptors. This finding suggests delayed photoreceptor development in patient ROs. Furthermore, AAV2.7m8-mediated *RS1* gene augmentation therapy rescued this phenotype, highlighting its potential therapeutic effect.

## Methods

### hiPSC lines generation and culture

The peripheral blood mononuclear cells (PBMCs) were isolated from peripheral blood using Lymphoprep (STEMCELL Technologies: #07801) through gradient centrifugation. Cells were cultured in StemSpan Serum-Free Expansion Medium II (SFEM II, STEMCELL Technologies, # 09605) along with CD34 Expansion Supplement (STEMCELL Technologies, # 02691) for four days. Subsequently, PBMCs were reprogrammed into hiPSCs using the CytoTune-iPS 2.0 Sendai Reprogramming Kit (Invitrogen, A16517) following the manufacturer’s instructions for a feeder-free workflow. Once hiPSC clones appeared, several monoclonal cells were manually picked and individually seeded in a Matrigel-coated plate (Corning, 354,277). hiPSCs were cultured in mTeSR Plus medium (STEMCELL Technologies, #100–0276) at 37℃ with 5% CO_2_. The medium was changed daily, and hiPSCs were passaged every 4–5 days using ethylenediaminetetraacetic acid (EDTA, Cellapy Biotechnology) at a 1:10 − 1:20 ratio.

### Three germ layers differentiation assay

Endoderm and mesoderm differentiation methods were adapted from a previously published study with slight modifications [36]. hiPSCs were dissociated into single cells using Accutase (Millipore, A6964) after reaching 80% confluency. Cells were then suspended in mTeSR Plus medium and treated with 20 µM Y27632 (Sigma, Y0503) at day 0. Cells were then cultured in mTeSR Plus medium for three days, during which the embryoid body (EB) was formed. Subsequently, EBs were seeded in a Matrigel-coated plate. For endoderm differentiation, EBs were cultured in an endoderm-induced medium (EIM) containing Dulbecco’s Modified Eagle Medium/Nutrient Mixture F-12 (DMEM/F12, Gibco), 20% fetal bovine serum (FBS, Gibco), 2 mM glutamine (Gibco), 0.1 mM β-mercaptoethanol (Gibco), 1X non-essential amino acids (NEAA, Gibco), and 1X penicillin-streptomycin (Gibco) for 3 weeks. For mesoderm differentiation, EBs were cultured in EIM supplemented with 100 µM ascorbic acid (Sigma) for 3 weeks. The STEMdiff Trilineage Differentiation Kit (STEMCELL Technologies, # 05230) was used according to its manufacturer’s ectoderm differentiation protocol.

### Genomic DNA extraction and DNA sequencing

Genomic DNA was extracted using a One-step cell genotyping kit (YSY Biotech) according to the manufacturer’s protocol. DNA sequencing was performed by Tsingke Biotechnology Co., Ltd. (Beijing, China). The primer sequences are listed in Supplemental Table 1.

### Induction of hiPSC differentiation into ROs

The differential method of ROs was based on a previously described method with slight modifications [37–39]. No bone morphogenetic protein-4 (BMP4) or retinoic acid (RA) was added during the whole culture process of ROs. Briefly, hiPSCs were dissociated into single cells by Accutase and suspended in a T25 bottle with mTeSR Plus medium and 20 µM Y27632 on day 0, at which time aggregates formed. To induce EBs formation, aggregates were cultured in a mixture of mTeSR Plus and neural induction medium (NIM) containing DMEM/F12, 1% N2 supplement (Gibco, A1370701), and 2 µg/ml heparin (Sigma), and then 1 × NEAAs and the mTeSR Plus medium were reduced proportionally on days 1–5. On day 6, EBs were seeded in a Matrigel-coated 60-mm petri dish and cultured with NIM until day 15. On day 16, the medium was changed to retinal differentiation medium (RDM) containing DMEM/F12 (3:7), 2% B27 supplement (Gibco, 17,504,044), 1 × NEAAs, and penicillin/streptomycin. Between days 20 and 28, loosely adherent neural retina domains exhibiting a golden-circle appearance were isolated using a 1 ml syringe needle. These isolated domains were then cultured in RDM until day 34. During this culture period, the isolated domains self-organized into three-dimensional ROs. Only the ROs that did not retain the golden-like appearance were retained for further study. On day 90, the medium was changed to the retinal culture medium 1 (RC1) containing DMEM/F12 (3:7), 10% FBS (Gibco), 2% B27 supplement, 100 mM Taurine (Sigma), 2 mM GlutaMAX (Gibco), 1 × NEAAs, and penicillin/streptomycin. For long-term culture, the medium was changed to the retinal culture medium 2 (RC2) containing DMEM/F12 (1:1), 10% fetal bovine serum (FBS, Gibco), 1% N2 supplement, 100 mM Taurine, 2 mM GlutaMAX, 1 × NEAAs, and penicillin/streptomycin. The workflow of RO generation is shown in Supplemental Fig. 1A.

### Overexpression of RS1 protein in HEK293T cells

HEK293T cells were seeded in a six-well plate and cultured in a maintenance growth medium (MGM) containing DMEM (Gibco), 10% FBS, and penicillin/streptomycin at 37℃ with 5% CO_2_. pLenti-RS1-eGFP and pLenti-RS1(E72K)-eGFP plasmids (General BIOL, China) were individually transfected into HEK293T cells using the Lipofectamine 3000 transfection kit (Invitrogen, L3000075) following the manufacturer’s instructions. After transfection, cells were cultured in MGM. However, cells used to assay the protein in the medium were cultured in MGM without FBS.

### Western blotting (WB)

For sodium dodecyl-sulfate polyacrylamide gel electrophoresis (SDS-PAGE) WB, HEK293T cells were lysed in radioimmunoprecipitation assay (RIPA) lysis buffer (Beyotime, P0013C). To analyze the octamer of RS1, HEK293T cells were lysed in cell lysis buffer for WB and immunoprecipitation (Beyotime, P0013). Cell lysates were centrifuged at 14,000 g for 15 min at 4 °C and the supernatant was collected. For secreted protein, the supernatant of the cell culture medium was centrifuged at 4,000 g for 60 min at 4℃ using Amicon Ultra-15 Centrifugal Filter Devices (Merck). Protein concentrations were determined by bicinchoninic acid (BCA) assay using the BCA Protein Assay Kit (Solarbio, PC0020). Protein under reducing conditions was boiled for 5 min before being mixed with 5× SDS-PAGE sample loading buffer (Beyotime, P0015). Afterward, the mixture was loaded onto 10% BeyoGel SDS-PAGE precast gel (Beyotime, P0052B). The protein under non-reducing conditions was mixed with 5× native gel sample loading buffer (Beyotime, P0016N), followed by loading onto 4–20% BeyoGel Plus Precast PAGE Gel (Beyotime, P0523M). After electrophoresis, samples were transferred onto a nitrocellulose membrane. Membranes were blocked with 5% bovine serum albumin (BSA, Solarbio, A8020) in Tris-buffered saline with 0.1% Tween 20 (TBST, Biosharp, BL602A) for 60 min and then incubated at 4 °C overnight with primary antibodies against RS1 at 1:2000 dilution in TBST with 5% BSA. Membranes were then washed thrice with TBST for 5 min each time, incubated with IRDye 800CW secondary antibodies at 1:5000 dilution (LI-COR, 926-32210) in TBST with 5% BSA for 60 min, and finally washed thrice with TBST for 5 min each time. Then immunoreactive proteins were detected using the Bio-Rad ChemiDoc Imaging System.

### Immunofluorescence (IF) staining

ROs were fixed in 4% paraformaldehyde in phosphate-buffered saline (PBS) for 20 min and dehydrated with 20% sucrose in PBS at 4℃ overnight before being embedded in an optimal cutting temperature compound (Thermo). Next, ROs were cryosectioned into a 10-micron section. The cryosections were blocked and permeated in 3% BSA, 5% goat serum, and 0.5% Triton X-100 in PBS at room temperature for 1 h before incubation with primary antibodies at 4℃ overnight. Cryosections were washed thrice with PBS and then incubated with the secondary antibodies at room temperature for 1 h. Finally, the sections were incubated with 4′,6-diamidino-2-phenylindole (DAPI) for nuclear staining. HEK293T cells were fixed for 20 min and stained for IF analysis. The stained sections and cells were photographed and analyzed using a confocal microscope (ZEISS LSM880). Antibodies used are listed in Supplemental Table 2.

### RNA extraction and quantitative real-time polymerase chain reactions (qPCR)

Total RNA was extracted using TRIzol (Invitrogen), and complementary DNA (cDNA) was synthesized using the HiScript II 1st Strand cDNA Synthesis Kit (Vazyme) according to the manufacturer’s protocol. Synthesized cDNAs were mixed with SYBR qPCR Master Mix (Vazyme) according to the manufacturer’s protocol. Afterward, the mixture was subjected to qPCR in a Roche LightCycler. All samples were normalized against the housekeeping gene glyceraldehyde 3-phosphate dehydrogenase (GAPDH) in each set of experiments, with at least three independent biological and three technical replicates of ROs. Each biological replicate contained 15–20 ROs. The relative gene expression was quantified using the 2^−∆∆Ct^ method. The primers were synthesized by GenScript (Nanjing, China), and the sequences are listed in Supplemental Table 1.

### RNA-seq analysis

ROs were suspended in 1 ml TRIzol at day 90 and stored at -80℃. Patient-1 and control-1 groups contained three independent biological replicates respectively. Each biological replicate contained 15–20 ROs. RNA extraction, library construction, and RNA-seq were performed by BGI Tech Solutions Co., Ltd. (Shenzhen, China). RNA-seq was performed on the BGISEQ platform. The sequencing length was paired-end (PE) 150. The raw reads were filtered using SOAPnuke and then stored in a FASTQ format. The hierarchical indexing for spliced alignment of transcripts (HISAT) was used to align the clean reads to the reference genome (GCF_000001405.39_GRCh38.p13), and the clean reads were aligned to the reference genes by Bowtie2. Next, the expression levels of samples were calculated using the RNA-Seq by Expectation-Maximization (RSEM). DEseq2 was used to analyze the differentially expressed genes (DEGs), using |log2 Fold Change| ≥1 and adjusted P -value (Q-value) ≤ 0.05 as the selection criteria. Based on the results of differential gene detection, hierarchical clustering analysis was performed on the concatenated differential genes using the pheatmap package in R.

### AAV production and treatment

*RS1* gene augmentation pAAV-CMV-RS1-HA-polyA and mCherry control pAAV-CMV-mCherry-polyA vectors were synthesized by GenScript. The two vectors were encapsulated into AAV2.7m8 by GENECHEM (Shanghai, China). On day 0, the ROs on day 70 were individually placed in each well of 96-well U-bottomed low attachment plates (Corning) and cultured in 100 µl of RC1. Additionally, 5E + 9 virus genomes were added to each well. On day 2, an extra 100 µl of RC1 was added. On day 4, the ROs with the old culture medium were transferred to 24-well low attachment plates (Corning), and another 500 µl of fresh RC1 was added. After 6 days of AAV exposure, the medium was replaced with a fresh RC1 medium on day 7.

### Microelectrode array (MEA) recording

Action potentials were recorded using MaxOne (MaxWell Biosystems, Switzerland). The RO was cut in half using scissors, and one part was planted in the MaxOne chip at week 8. The RO on the chip was cultured in the RC1 medium. Before planting, the chip was treated with 1% Terg-a-zyme (Sigma) and coated with 0.07% polyethylenimine (Sigma) and 0.04 mg/mL of laminin (Sigma, L2020). The chip is a high-density microelectrode array with an 8 mm [2] sensor area and 26,400 platinum electrodes. MaxLab Live Software (MaxWell Biosystems, Switzerland) was used to record and analyze the action potentials. An electrode was considered active if it had a firing rate larger than 0.1 Hz and a spike amplitude greater than 20 µV.

### Transmission electron microscopy (TEM)

RO samples were fixed in an electron microscope fixative (Servicebio, G1102) at 4℃ for 2 h and then rinsed thrice in 0.1 M phosphate buffer. They were postfixed in 1% osmium tetroxide and 0.1 M phosphate buffer at room temperature for 2 h, and subjected to gradient dehydration and infiltration. Subsequently, ultrathin sections were cut and stained. Images were photographed under TEM (HITACHI).

### Statistical analysis

Statistical analyses were performed using GraphPad Prism (GraphPad Prism 9). The unpaired Student’s t-test was utilized for two-group comparisons. In the statistical analysis of IF staining, each dot represented one biological replicate. *P* < 0.05 was considered statistically significant. ns (not significant) indicated *p* > 0.05, ∗ indicated *p* < 0.05, ∗∗ indicated *p* < 0.01, and ∗∗∗ indicated *p* < 0.001.

## Results

### Generation and characterization of hiPSCs derived from XLRS patients

**Fig. 1:**
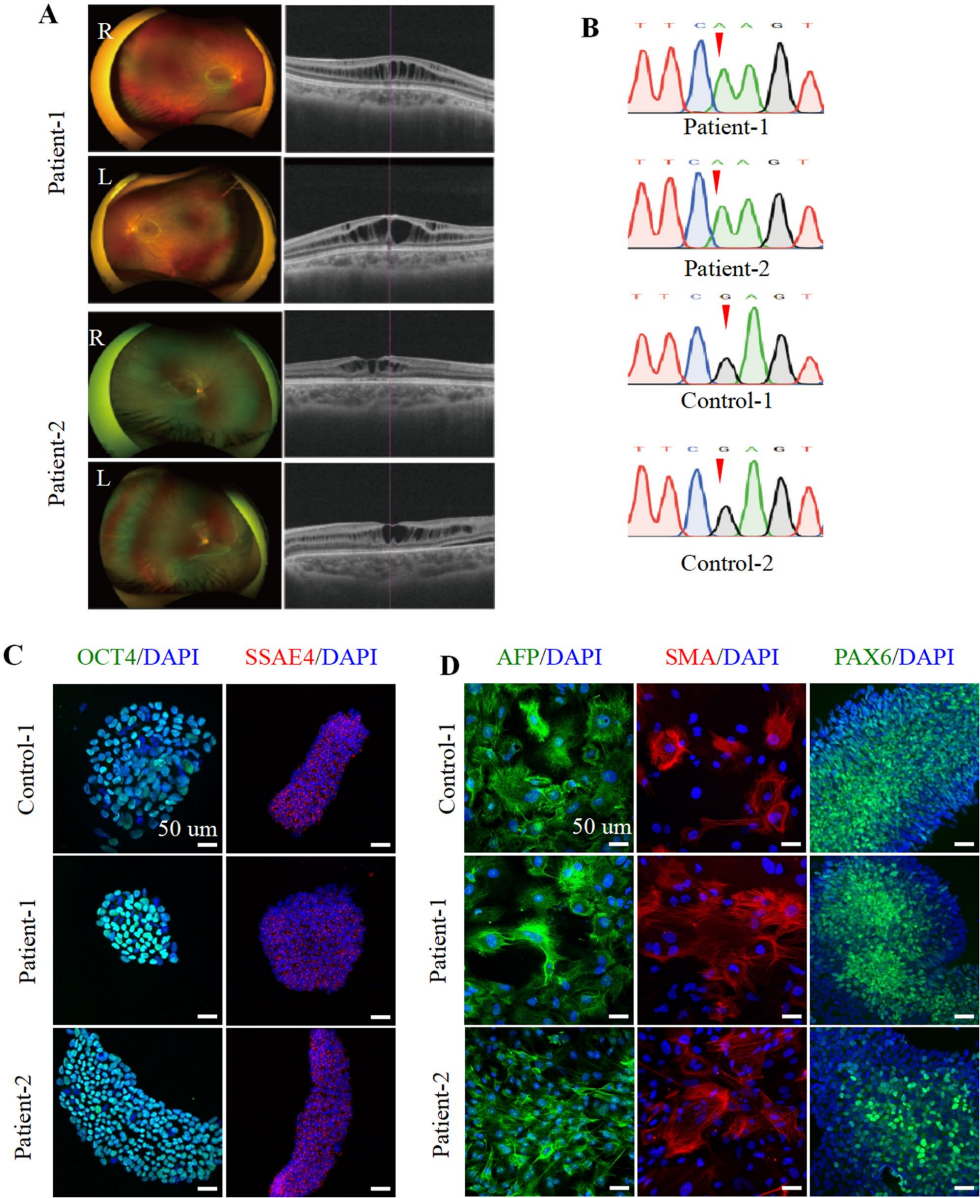
Generation and characterization of hiPSC lines from XLRS patients harboring RS1 mutation (E72K). (**A**). SLO and OCT of patinet-1 and patient-2. (**B**). DNA sanger sequencing of hiPSC of patients and controls. (**C**). Immunofluorescence staining of pluripotent stem cell markers OCT4 and SSEA4 in control-1, patient-1, and patient-2. Scale bar, 50 μm. (**D**). Immunofluorescence staining of endodermal marker (AFP), mesodermal marker (SMA), and ectodermal marker (PAX6) in control-1, patient-1, and patient-2. Scale bar, 50 μm. (**C**-**D**). Nuclei were stained by DAPI (blue)

Two patients diagnosed with X-linked retinoschisis (XLRS), designated as patient-1 and patient-2, along with two healthy controls identified as control-1 and control-2, were enrolled in the study. PBMCs from patient-1, patient-2, and control-1 were reprogrammed into hiPSC lines. Control-2 iPSCs were acquired from Nuwacell Biotechnology (Hefei, China). Comprehensive clinical profiles and reprogramming information are listed in Table S3. Scanning laser ophthalmoscopy (SLO) and optical coherence tomography (OCT) revealed typical macular schisis in both retinas of the XLRS patients. Patient-1 exhibited the schisis localized within the inner nuclear layer (INL) and inner plexiform layer (IPL), while patient-2’s schisis was confined solely to the INL (Fig. 1A). Control-1 presented ordinary fundus features and OCT morphology (Figure S1B). The generated hiPSC lines displayed typical stem cell characteristics, characterized by tightly clustered colonies and a high nucleocytoplasmic ratio (Figure S1C). hiPSCs derived from patient-1 and patient-2 exhibited a mutation in exon 4 of the *RS1* gene (c.214G > A), while control-1 and control-2 hiPSCs displayed wild-type *RS1* sequences (Fig. 1B). IF staining confirmed the expression of pluripotent stem cell markers OCT4 and SSEA4 in hiPSCs from control-1, patient-1, and patient-2 (Fig. 1C). Moreover, their multilineage differentiation potential was validated through the expression of lineage-specific markers, AFP for endoderm, SMA for mesoderm, and PAX6 for ectoderm (Fig. 1D). Moreover, all hiPSC lines had normal karyotypes (Figure S1D). Collectively, these results demonstrated the successful establishment of patient and control iPSC lines, which are vital for OR generation.

### hiPSC-derived laminated ROs mimic the human retina

**Fig. 2:**
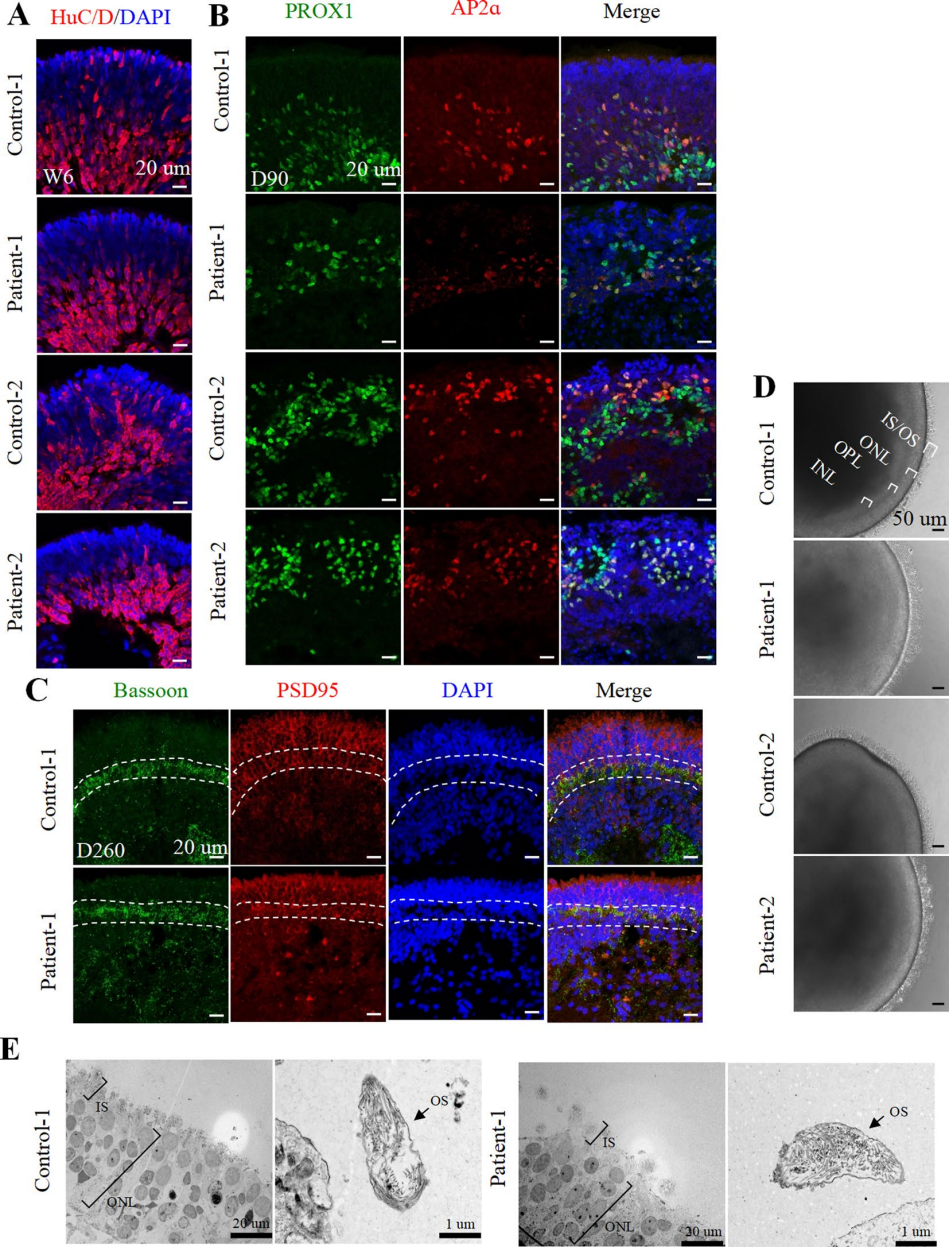
The ROs mimic the human retina after long-term culture. (**A**). Immunofluorescence staining of presumptive ganglion cells and/or amacrine cells marker HuC/D in ROs at week 6. Scale bar, 20 μm. (**B**). Immunofluorescence staining of horizontal cell marker Prox1 and the amacrine cell marker AP2α and PROX1 in ROs at week 6. Scale bar, 20 μm. (**C**). Representative immunofluorescence staining images of ribbon synapse proteins Bassoon and postsynaptic proteins PSD95 in control-1 and patient-1 iPSC-derived ROs at day 260. Scale bar, 20 μm. (**A**-**C**). Cell nuclei were stained with DAPI (blue). (**D**). Bright-field images of the ROs at day 220. Scale bar, 50 μm. (**E**). The TEM images of control-1 and patient-1 iPSC-derived RO at day 220. Scale bar of the left panel in control-1 and patient-1, 20 μm. Scale bar of the right panel in control-1 and patient-1, 10 μm

To establish an in vitro disease model, hiPSCs obtained from patient-1, patient-2, control-1, and control-2 were utilized for RO construction. Both patient and control iPSC-derived ROs exhibited the expression of eye-field transcription factors PAX6, retinal progenitor cell markers CHX10, and OTX2 during the early stages of development (Figure S2A-B). On week 6, ROs expressed presumptive ganglion cells and/or amacrine cell marker HuC/D (Fig. 2A). Throughout development, both control and patient iPSC-derived ROs displayed similar expression patterns of the horizontal cell marker PROX1 and the amacrine cell markers AP2α and PROX1 (Fig. 2B). With the long-term culture of ROs, the ribbon synapse protein Bassoon was observed juxtaposed with the postsynaptic protein postsynaptic density protein 95 (PSD95) in control-1 and patient-1 iPSC-derived ROs at day 260 (Fig. 2C). Further observation under inverted microscopy revealed that ROs exhibited a laminated neuroretinal structure with a brush-like border over time. On day 220, the multilayered shape became easily distinguishable, with the inner nuclear layer (INL), the outer plexiform layer (OPL), the outer nuclear layer (ONL), and the inner segment and outer segment (IS/OS) of the photoreceptor observed in mature ROs, mimicking the human retina (Fig. 2D). Moreover, TEM confirmed the ultrastructure of the brush-like structure at the apical of ROs, validating the formation OS (Fig. 2E). Collectively, these data indicate that RO generation using this method mimicked the human retina, making it a suitable in vitro XLRS disease model.

### Mutant RS1 (E72K) leads to defective RS1 protein

**Fig. 3:**
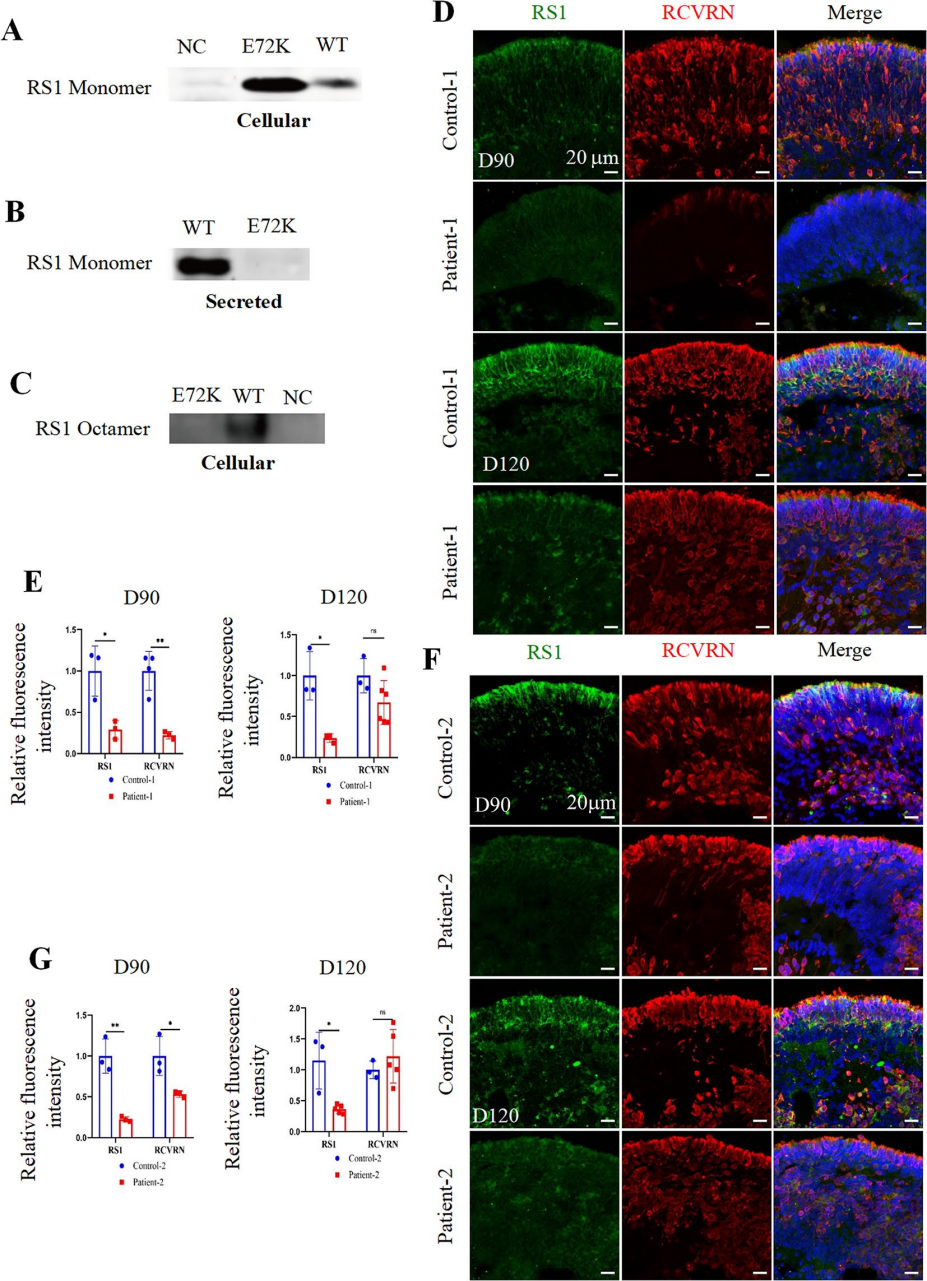
*In vitro* expression of RS1 (E72K). (**A**). Western blotting of RS1 in HEK293T overexpression cells under reducing conditions. (**B**) Western blotting of RS1 in HEK293T overexpression cell culture medium under reducing conditions. (**C**) Western blotting of RS1 in HEK293T overexpression cells under non-reducing gel conditions. (**D**, **F**). Representative immunofluorescence staining images of RS1 and photoreceptor-specific marker recoverin (RCVRN) in the control-1, patient-1, control-2, and patient-2 ROs at day 90 and day 120, separately. Cell nuclei were stained with DAPI (blue). Scale bar, 20 μm. (**E**, **G**). Quantification of the relative fluorescence intensity of RS1 and RCVEN in the control-1, patient-1, control-2, and patient-2 ROs at day 90 and day 120, separately

To assess the impact of the c.214G > A mutation on RS1 secretion or octamer formation, wild-type (WT) and RS1 (E72K) mutation proteins were overexpressed in HEK293T cells. Subsequently, cell lysates and cell medium supernatants were collected. WB under reducing conditions revealed that the RS1 (E72K) mutation manifested as monomers in cell lysates, similar to WT RS1 (Fig. 3A). However, RS1 (E72K) mutation was not detected in the cell medium supernatants compared with the WT RS1 (Fig. 3B). Further investigation into the effect of the RS1 (E72K) mutation on octamer formation was conducted through non-reducing WB analysis. The WT RS1 was resolved at a size of about 180 kDa, corresponding to the dimension of an octameric complex. However, the band was not detected in the RS1 (E72K) mutation lane (Fig. 3C). The full-length blots are presented in Figure S3A-B.

To investigate whether the RS1 (E72K) mutation disrupts the protein secretion pathway, we analyzed the co-localization of RS1 with specific markers in overexpressed HEK293T cells. These markers included GRP94 and Golgi97. Confocal fluorescent microscopy revealed colocalization of the RS1 (E72K) mutation with GRP94 in the ER and Golgi97 in the Golgi complex, consistent with the WT counterpart (Figure S3D), suggesting that the RS1 (E72K) mutation could reach both the ER and Golgi complex.

Next, IF staining in RO cryosections was conducted to further explore the expression pattern of RS1 (E72K) mutation. The expression of RS1 was detected in control-1 and control-2 ROs as early as day 90, whereas it was virtually absent in patient-1 and patient-2. Throughout RO development, RS1 expression consistently increased over time in both the patient and control ROs. However, RS1 expression was significantly reduced in patient-1 and patient-2 ROs compared to control-1 and control-2 ROs (Fig. 3D-G).

Our findings collectively suggest that the RS1 (E72K) mutation disrupts the protein’s functionality in two ways: Impaired Octamer Formation: the mutation prevents the RS1 protein from assembling into functional octameric complexes within cells. Secretion Deficiency: the mutated protein appears to be unable to be secreted out of the cells. Although RS1 protein was observed in *RS1* mutant (c.214G > A) ROs, its expression was decreased compared to control ROs.

### Delayed development of photoreceptor cells in RS1 (E72K) mutation ROs

**Fig. 4:**
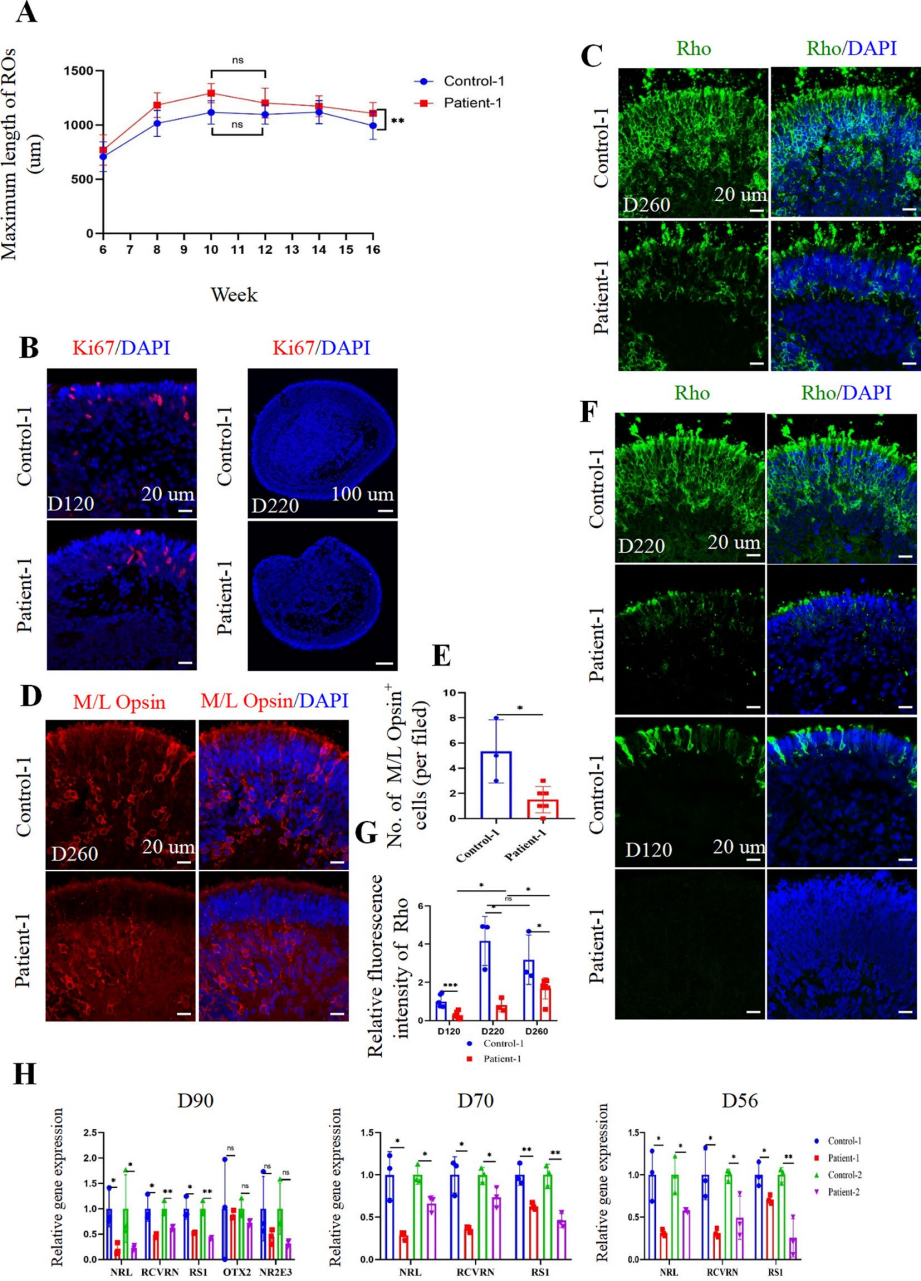
The development of ROs. (**A**). The maximum length of control-1 and patient-1 iPSC-derived ROs from week 6 to week 16. (**B**). Representative immunofluorescence staining images of mitotic marker Ki67 in the control-1 and patient-1 iPSC-derived ROs at day 220 and 260. Scale bar of the left panel, 20 μm. Scale bar of the right panel. 100 μm. (**C**, **D**). Representative immunofluorescence staining of rhodopsin (Rho)-positive rod photoreceptor (C) and M/L Opsin-positive cone photoreceptor (**D**) in control-1 and patient-1 iPSC-derived ROs at day 260. Scale bar, 20 μm. (**E**). Quantification of M/L Opsin positive cell density in control-1 and patient-1 iPSC-derived ROs at day 260. (**F**). Representative immunofluorescence staining images of Rho in the control-1 and patient-1 iPSC-derived ROs at day 220 and day 120. Scale bar, 20 μm. (**B**-**D**, **F**). The cell nuclei were stained with DAPI (blue). (**G**). Quantification of relative fluorescence intensity of Rho in the control-1 and patient-1 iPSC-derived ROs at day 120, day 220, and day 260. (**H**). qPCR analysis of rod-specific precursor gene NRL, photoreceptor-specific gene RCVRN, photoreceptor fate gene OTX2, cone photoreceptor gene NR2E3, and RS1 in the control-1, patient-1, control-2, and patient-2 ROs at day 56 to day 90, separately

To evaluate the development of ROs, the maximum length of ROs and the expression of the mitotic marker Ki67 were assessed. The growth of ROs’ maximum length persisted until week 10, indicating immature RO (Fig. 4A). On day 120, both control-1 and patient-1 iPSC-derived ROs exhibited Ki67 expression. However, on day 220, Ki67-labeled cells in both control-1 and patient-1 iPSC-derived ROs were scarce (Fig. 4B), suggesting a gradual cessation of proliferation and progression toward maturity. We investigated the retinal cell classes of ROs at day 260. There was no significant difference in GS and Sox9-positive Müller glial cells between control-1 and patient-1 iPSC-derived ROs at day 260 (Figure S4A-C). Besides, the cell density of PKCɑ-positive rod bipolar cells and Go(ɑ)-positive ON bipolar cells did not significantly differ between control-1 and patient-1 iPSC-derived ROs at day 260 (Figure S4D-E). At day 260, the expression levels of rhodopsin (Rho) and M/L Opsin were decreased in patient-1 iPSC-derived ROs compared with control-1 iPSC-derived ROs (Fig. 4C-E, G and Figure S4F). To investigate whether these differences were attributed to the delayed maturation of RS1 (E72K) mutation ROs, we reviewed the expression of Rho in control-1 and patient-1 iPSC-derived ROs at days 120–220 (Fig. 4F, G). Significantly lower Rho expression was observed in patient-1 iPSC-derived ROs compared to control-1 iPSC-derived ROs during this period. Furthermore, Rho expression increased in patient-1 iPSC-derived ROs from days 120–260, whereas it remained stagnant in control-1 iPSC-derived ROs after day 220. To investigate whether the deficiency of Rho in patient-1 iPSC-derived ROs resulted from cell death, Caspase-3 IF staining was performed. Results showed no significant difference between control-1 and patient-1 iPSC-derived ROs at day 120 (Figure S4G-H), suggesting that photoreceptor deficiency in *RS1* mutation ROs was unrelated to apoptosis.

To further investigate the effects of the RS1 (E72K) mutation on the RO development even before photoreceptor maturation, we analyzed proteins and genes associated with photoreceptor precursor cells. On day 90, the gene and protein expression of rod-specific precursor marker NRL and photoreceptor-specific marker recoverin (RCVRN) werereduced in the patient ROs, alongside decreased RS1 expression (Fig. 4H, Figure S4I-J and Fig. 3D-G). However, no significant differences were observed in gene expression levels of the photoreceptor fate marker OTX2 and cone photoreceptor fate marker NR2E3 between the control and patient ROs (Fig. 4H). Furthermore, gene expression levels of NRL and RCVRN, specific markers of photoreceptor precursor cells, were reduced in patient ROs at even earlier time points (days 70 and 56). This finding was consistent with the decreased expression of RS1 (Fig. 4H).

In summary, these results indicate the crucial role of RS1 in photoreceptor development, with the RS1 (E72K) mutation causing a delay in photoreceptor maturation, particularly in the Rho subtype. Moreover, this developmental delay may be attributed to alterations in NRL and RCVRN expression preceding photoreceptor maturation.

### Altered spontaneous activity in RS1 (E72K) mutation ROs

**Fig. 5:**
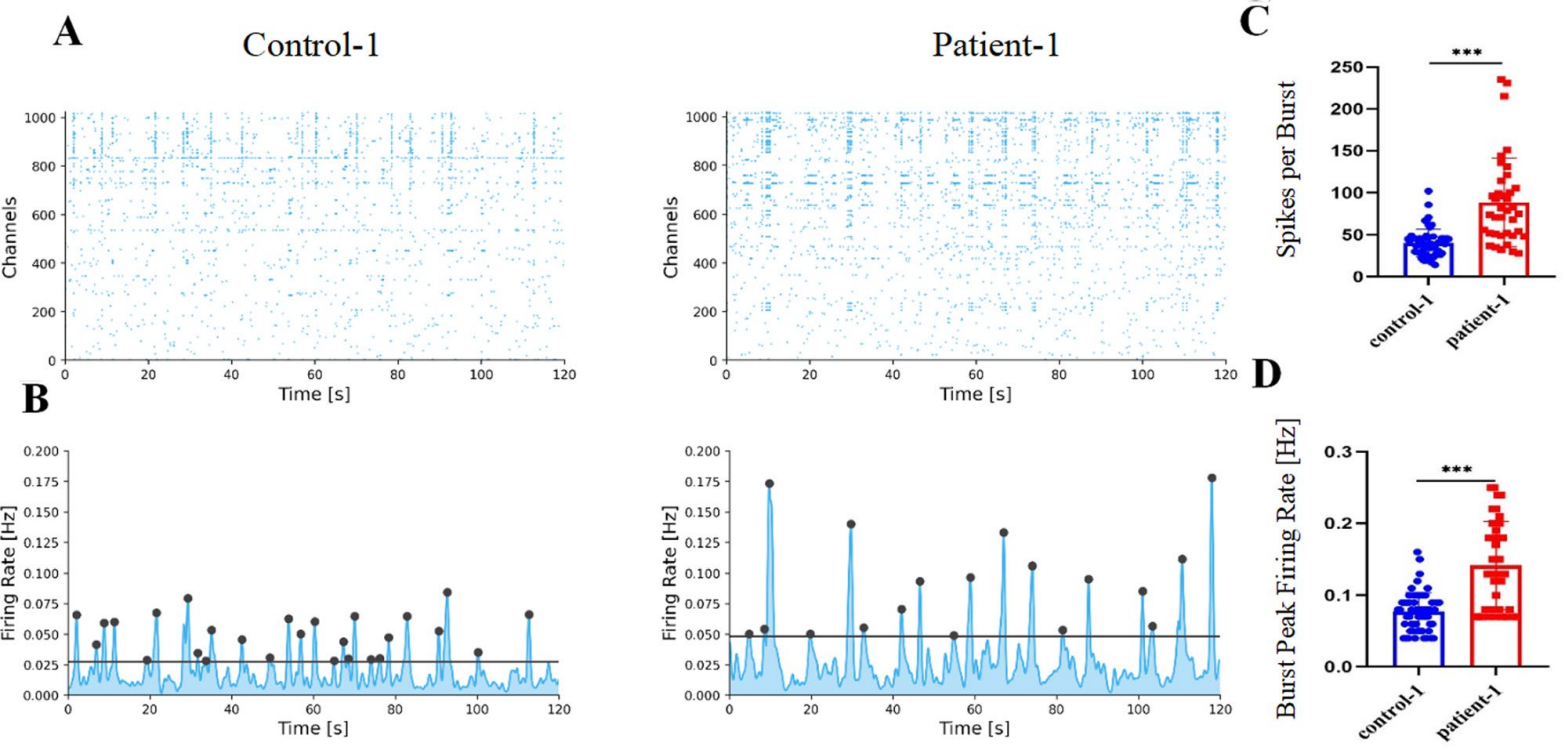
Spontaneous activity in ROs. (**A**). The raster plot of control-1 and patient-1 iPSC-derived RO after culture in the chip for 3 weeks. (**B**) Network activity plot of control-1 and patient-1 iPSC-derived RO after culture in the chip for 3 weeks. (**C**). Quantification of spikes per burst in control-1 and patient-1. (**D**). Quantification of burst peak firing rate in control-1 and patient-1

To determine the electrophysiological function of the retinal ganglion cell (RGC) in immature ROs, the spontaneous activity of control-1 and patient-1 iPSC-derived RO was assessed using a high-density MEA. The ROs were placed on the chip at week 8 and analyzed at week 12. Raster and network activity plot displayed spiking activities at each channel for 120 s. Rhythmic bursting activities recorded by MEA confirmed the reliability of spontaneous activity results in both control-1 and patient-1 iPSC-derived RO (Fig. 5A-B). The spikes within bursts in control-1 iPSC-derived ROs were observed at a rate of 42.9%, while in patient-1 iPSC-derived ROs, it was 37.64%. The spikes per burst were increased in patient-1 iPSC-derived RO compared with control-1 iPSC-derived RO (88.79 ± 51.86 versus 40.25 ± 16.21). Similarly, the burst peak firing rate was higher in patient-1 iPSC-derived ROs compared to control-1 iPSC-derived RO (0.14 ± 0.06 Hz versus 0.08 ± 0.03 Hz) (Fig. 5C-D).

These results demonstrate an increased spontaneous activity in *RS1* (E72K) mutation ROs, suggesting a potential effect on RGC activity in *RS1*-mutant ROs during the immature period.

### Gene expression changes associated with RS1 (E72K) mutation in ROs

**Fig. 6:**
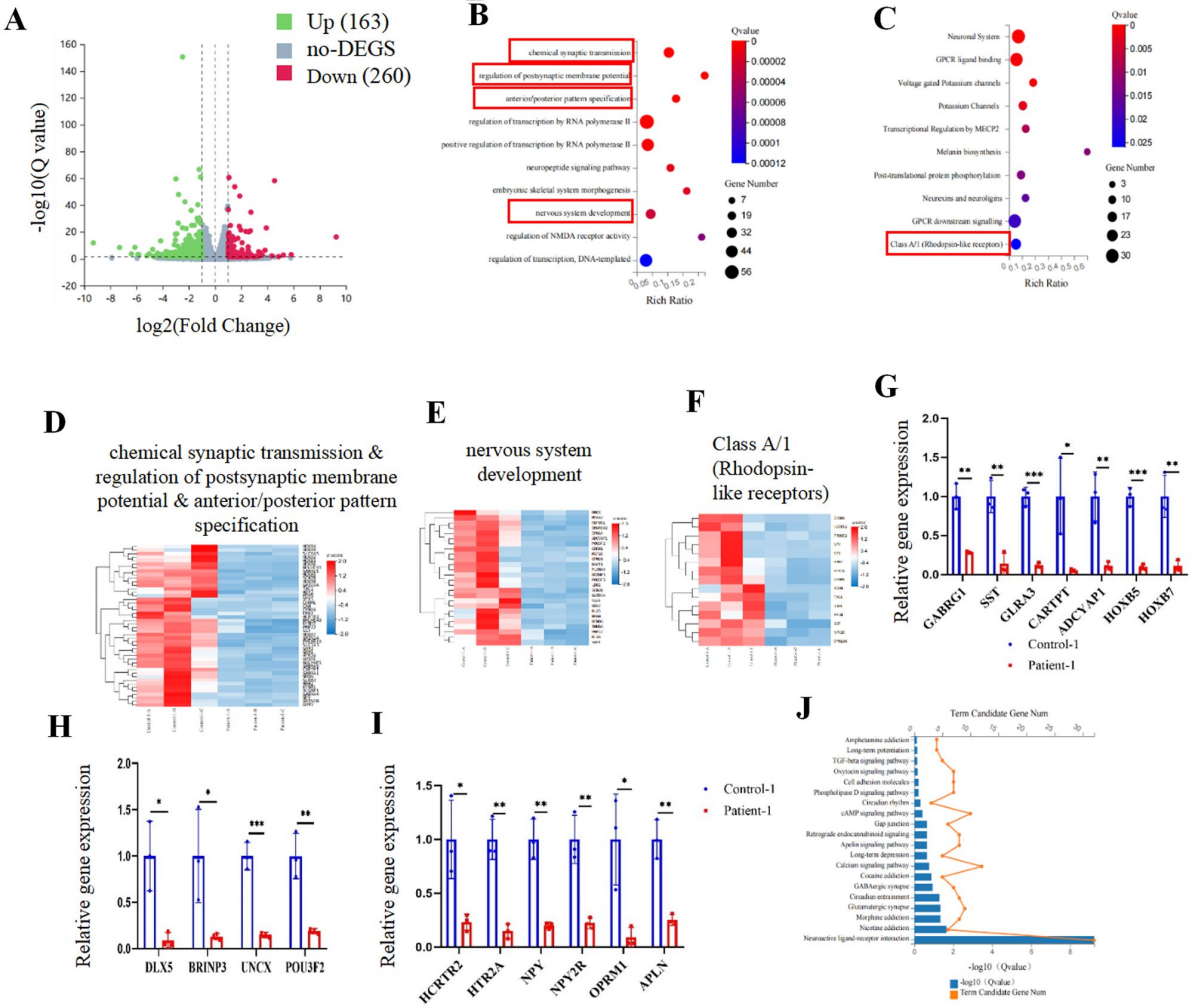
RNA-seq analysis of samples from the control-1 and patient-1 iPSC-derived ROs at day 90. (**A**). The volcano plot of RNA-seq showing the upregulated and downregulated genes in patient-1 iPSC-derived ROs at day 90. (**B**). The bubble plot of the top 10 significant biological processes (BPs) of Gene Ontology (GO) for the downregulated genes. (**C**). The bubble plot of the top 10 significant Reactome terms of the downregulated genes. (**D**). The heat map of differentially expressed genes (DEGs) in chemical synaptic transmission, regulation of postsynaptic membrane potential, and anterior/posterior pattern specification. (**E**). The heat map of DEGs in nervous system development. (**F**). The heat map of DEGs in Class A/1 (Rhodopsin-like receptors). (**G**-**I**). The validation of genes in panel d (**G**), panel e (**H**), and panel f (**I**) by qPCR analysis. (**J**). The histogram of the top 10 significant pathways in the Kyoto Encyclopedia of Genes and Genomes (KEGG) for patient-1 iPSC-derived ROs.

RNA-seq was performed on control-1 and patient-1 iPSC-derived ROs at day 90 to explore the transcriptomic change between ordinary and RS1 (E72K) mutant ROs. Principal component analyses (PCA) and the Pearson correlation coefficient demonstrated robust biological reproducibility among samples from control-1 and patient-1 (Figure S5A-B). The volcano plot revealed that 163 genes were upregulated and 260 genes were downregulated in patient-1 compared to control-1 (Fig. 6A). Next, the top 10 Gene Ontology (GO) terms of significantly downregulated and upregulated genes were performed separately to delve into differences in biological process (BP) between these two groups. Notably, GO BPs of downregulated genes were related to chemical synaptic transmission, regulation of postsynaptic membrane potential, anterior/posterior pattern specification, and nervous system development (Fig. 6B). Furthermore, reactome enrichment analysis revealed that DEGs were associated with Class A/1 (Rhodopsin-like receptors) (Fig. 6C). The DEGs within these signaling pathways are presented in Fig. 6D-F. GO BPs of upregulated genes are presented in Figure S5C. Validation of RNA-seq findings was performed via qPCR assays. Genes associated with chemical synaptic transmission, regulation of postsynaptic membrane potential, anterior/posterior pattern specification (e.g., GABRG1, SST, GLRA3, CARTPT, ADCYAP1, HOXB5, and HOXB7), and nervous system development (e.g., DLX5, BRINP3, UNCX, and POU3F2) were significantly downregulated (Fig. 6G-H). Furthermore, Class A/1 (Rhodopsin-like receptors)-related genes (e.g., HCRTR2, HTR2A, NPY, NPY2R, OPRM1, and APLN) were also markedly downregulated (Fig. 6I). The Kyoto Encyclopedia of Genes and Genomes (KEGG) pathway enrichment analysis unveiled significant enrichment of DEGs in pathways such as gap junction, calcium signaling, GABAergic synapse, glutamatergic synapse, and neuroactive ligand-receptor interaction (Fig. 6J).

These findings collectively suggest a downregulation of genes associated with photoreceptors and synapses in *RS1* mutation ROs as early as day 90. This downregulation can potentially influence photoreceptor development.

#### Delayed development of photoreceptor cells in RS1(E72K) mutant ROs rescued by AAV2.7m8-mediated RS1 supplement

**Fig. 7:**
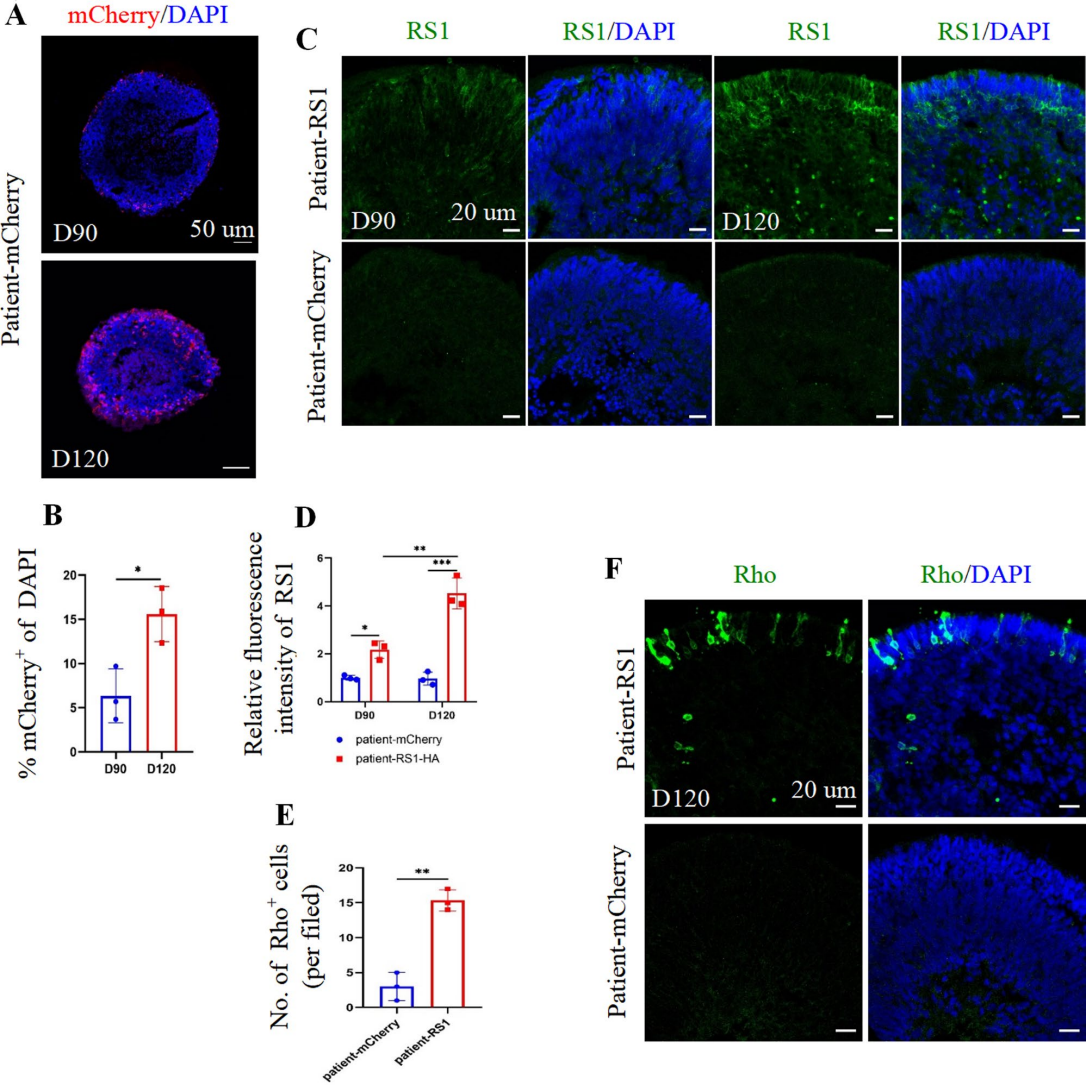
AAV2.7m8 transfection rescued photoreceptor development delay by ***RS1*** augmentation therapy. (**A**). The mCherry in patient-mCherry RO sections at day 90 and day 120. Scale bar, 50 μm. (**B**). Quantification of the percentages of mCherry over DAPI in patient-mCherry ROs sections at day 90 and day 120. (**C**). Representative immunofluorescence staining images of RS1 in patient-RS1 and patient-mCherry ROs at day 90 and day 120. Scale bar, 20 μm. (**D**). Quantification of relative fluorescence intensity of RS1 in patient-RS1 and patient-mCheery ROs at day 90 and day 120. (**E**). The Rho-positive cells were quantified in patient-RS1 and patient-mCheery ROs at day 120. (**F**). Representative immunofluorescence staining images of Rho in patient-RS1 and patient-mCherry ROs at day 120. Scale bar, 20 μm. (A, CF). The cell nuclei were stained with DAPI (blue)

To investigate the effect of RS1 on photoreceptor development in ROs, AAV2.7m8-RS1 and AAV2.7m8-mCherry were individually transduced into patient-1 iPSC-derived ROs at day 70, prior to photoreceptor maturation. Following viral transduction, mCherry fluorescence was primarily detected in the outer nuclear layer of patient-derived AAV2.7m8-mCherry ROs. After transduction, mCherry was detected mainly in the outer layer of patient AA2.7m8-mCherry ROs. From days 90 to 120, the expression of mCherry increased, and the transduction efficiency increased from 6·35% ± 3·05% to 15·59% ± 3.11% (Fig. 7A-B). On day 90, RS1 was widely distributed in patient AAV2.7m8-RS1 ROs and exhibited high expression levels, as evidenced by immunofluorescence staining, in contrast to patient AAV2.7m8-mCherry ROs, indicating successful penetration of 5 + E9 vector genomes AAV2.7m8-RS1 into the ROs and expression of recombinant RS1 protein. The expression of recombinant RS1 in patient AAV2.7m8-RS1 ROs continued to increase at day 120 (Fig. 7C-D).

To investigate whether elevated RS1 influenced photoreceptor development in patient-1 iPSC-derived ROs, the expression of photoreceptor-related markers in ROs was examined. On day 90, the expression pattern of NRL in patient AAV2.7m8-RS1 ROs was increased compared to patient AAV2.7m8-mCherry ROs (Figure S6A-B), suggesting that AAV2.7m8-mediated RS1 gene augmentation accelerated photoreceptor development in a short time. On day 120, significant enhancement in Rho expression was observed via immunostaining in patient AAV2.7m8-RS1 ROs compared to patient AAV2.7m8-mCherry ROs (Fig. 7E-F). Furthermore, the rod photoreceptors in AAV2.7m8-RS1 ROs displayed morphology (shape and structure) closely resembling that of control-1 iPSC-derived ROs (Figs. 4F and 7F). This suggests that RS1 gene augmentation therapy promoted the development of rod photoreceptors in the ROs, potentially normalizing their structure.

In summary, these findings illustrate that AAV2.7m8-mediated RS1 supplementation in ROs partially restored delayed photoreceptor development.

## Discussion

The current knowledge about XLRS is partly derived from animal models. Although the phenotype presented in these models resembles that of XLRS patients, rodent and primate retinas vary significantly [26, 40–42]. In this study, hiPSCs with the *RS1* missense variant of c.214G > A (p.E72K), and a genetic background comparable to that of XLRS patients, were successfully constructed. hiPSC-derived ROs were utilized as an in vitro model to investigate the impact of *RS1* mutations on RO development and the therapeutic effects of *RS1* gene augmentation in early-stage ROs. The quality of the differentiated outcomes may be affected by the initial conditions of the iPSCs [43, 44]. Besides, the differentiation of ROs is a nonlinear process that takes a long time, increasing variability [45]. Therefore, using a single disease hiPSC line may not be sufficient to draw persuasive conclusions. Previous studies used genetic engineering technology to introduce mutation to hiPSCs to create a disease hiPSC line [46, 47]. In this study, we constructed two hiPSC lines with the same mutation from separate XLRS patient PBMCs, which increased the reliability of our results. Our study primarily focused on hiPSC lines patient-1 and control-1, with patient-2 and control-2 serving as supplemental descriptions.

For ROs to serve as reliable models for retinopathy diseases, they must accurately replicate the human retina. This includes recapitulating the specific cell types and disease-relevant phenotypes observed in patients. Previously, researchers have employed various methods utilizing different small molecules to achieve differentiation within ROs. There are subtle differences in ROs between different differentiation methods by regulating different pathways [46, 47, 48]. In the present study, with the long-term culture of ROs, retinal progenitor cells gradually differentiated into ganglion cells, amacrine cells, horizontal cells, photoreceptor cells, bipolar cells, and Müller glial cells, resembling human embryonic retina development [35]. In addition, the differentiated ROs reproduced a five-layer structure of the human retina, comprising the INL, OPL, ONL, IS, and OS. Moreover, synapses were formed at the late stage of RO differentiation. Overall, these results showed that the RO differentiation protocol we used was favorable.

The RS1 overexpression assay in HEK293 T cells showed that while the RS1 (E72K) mutation could be presented as a monomer within cells, it could not be secreted or form an octamer. These results are consistent with previous in vitro studies [49]. This indicates that the RS1 (E72K) mutation affects the folding of RS1. The RS1 (E72K) mutation showed colocalization with Golgi97 and GRP94, indicating that the E72K-mutant RS1 protein reached the Golgi complex and was retained intracellularly. These results, combined with those of IF staining of the RS1 (E72K) mutation in HEK293T cells, proved that the RS1 (E72K) mutation expression profile was rationalized on organoids. Our data also confirmed that the RS1 protein and *RS1* gene are significantly reduced but not missing in E72K mutant ROs. Therefore, using the RS1 (E72K) mutation RO to explore the relationship between the RS1 (E72K) mutation and retinal development is reasonable.

The discoidin domain of the RS1 protein, encoded by exon 4–6, contributes to the adhesive function of RS1 [50]. The RS1 (E72K) mutation, encoded by exon 4, affects this domain. The RS1 (E72K) mutation ROs did not develop a prominent splitting phenotype, even after extensive culture for 260 days. This finding contrasts with the retinal schisis observed in the XLRS patient. The following two possible explanations may explain this phenomenon: Delayed onset: the splitting phenotype in RS1 (E72K) mutations might manifest later in development, and the 260-day culture period might not have been sufficient to recapitulate this aspect of the disease. Subtle phenotypes: alternatively, the splitting phenotype caused by the mutation might be more subtle and require more sensitive methods for detection in the RO model. However, RS1 mutation (G488A and C625T) ROs exhibited splitting phenotypes and absent photoreceptor OS in a previous study [51]. As articles published before, XLRS manifests between infancy and school age with variable phenotypic presentation and without reliable genotype-phenotype correlations [5]. Even in our research, the phenotype did not look the same in two XLRS patients with the same RS1 mutation. The split was localized in the inner nuclear layer (INL) and the inner plexiform layer (IPL) in the patient-1 retina and only in the INL in the patient-2 retina. Considering this, we attribute the phenotypic discrepancy between our ROs and Shih-Hwa Chiou’s ROs to differences in the mutation sites [51]. When the XLRS patients with *RS1* mutation (c.214G > A) we recruited came to us for help, it was at their school age. Therefore, it is possible that the retinoschisis phenotype may not be observable under an inverted microscope at the current time, or it may require more culture time.

RO cell populations are considered to reach a stable, “developed” state around days 210 to 260 [32, 43]. In the this study, a negative expression of Ki67 at day 220 suggested that ROs tend to mature from day 220. Moreover, during the prolonged culture of ROs, the expression of Rho-positive rods in control-1 did not differ significantly between days 220 and 260. These results demonstrated that rod photoreceptor cells matured and had stable expression in control-1 iPSC-derived ROs at day 220. However, the expression of Rho increased persistently in RS1 (E72K) mutation ROs from days 220 to 260 and declined compared with control ROs. M/L opsin-positive cone photoreceptors were also decreased in RS1 (E72K) mutation ROs at day 260. These results, together with the deficient Rho expression at day 120, suggest that RS1 (E72K) mutant ROs delay the development of photoreceptor cells in ROs. Considering that RS1 was expressed at day 90, we hypothesized that RS1 may affect precursor photoreceptor cells at the early stage of ROs. Therefore, genes that regulate photoreceptor differentiation were investigated. NRL marks rod precursors and regulates rod photoreceptor cell occurrence and differentiation. RCVRN is involved in the visual phototransduction cascade. NRL^−/−^ gene-edited human embryonic stem cells generate rod-deficient ROs enriched in S-cone-like photoreceptors [52]. Wang et al. reported that postmitotic cone-committed precursors were in place in the outer retina before mid-gestation at embryonic day 50 (E50), and they were marked by loss of PAX6 and transient expression of RCVRN. NRL rod precursors did not appear until E65 [53]. The present study assessed the expression of NRL and RCVRN, and the results showed reduced expression of NRL and RCVRN in RS1 (E72K) mutation ROs from days 56 to 90. We inferred that RS1 may affect photoreceptor development by regulating NRL and RCVRN at the early stage of ROs. A recent study revealed that *RS1* mutations causes defective rod and cone photoreceptor cells in ROs [51]. However, our study demonstrated that photoreceptor cells were developmentally delayed at *RS1* mutation ROs. Besides the photoreceptor cells, we also identified Müller glial cells and bipolar cells, which are considered to be related to XLRS [54–56]. However, the number of Müller glial cells and bipolar cells did not differ significantly between patient and control ROs at day 260.

The rhythmic spontaneously bursting activity is generated in the immature retina due to the connectivity of RGCs and is observed in embryonic animals [57]. Rhythmic spontaneous bursting activities were observed in the current and the spikes per burst and burst peak firing rate were increased in patient-1 iPSC-derived RO at day 84. Hallam et al. reported that numerous RGCs from single and pooled ROs exhibited clear responses to light, 3′,5′-cyclic guanosine monophosphate (cGMP), and gamma-aminobutyric acid (GABA) stimulation, suggesting rudimental but functional retinal circuitry in both types of ROs [58]. A previous study also reported a significant increase in spontaneous firing rate in Rs1 mutant mice compared with WT controls. They considered that elevated activity acts as a barrier to visual signal discrimination [40]. Furthermore, the spontaneous activity observed before the onset of vision may be related to retinal projection to the central visual system and drive retinal developmental processes [59–61]. Therefore, we infer that the increased firing rate at an early stage of RS1 (E72K) mutation ROs might adversely affect retinal circuitry, synaptic remodeling, and retinal development.

Next, RNA-sequencing was performed on patient-1 and control-1 iPSC-derived ROs at D90 to further explore the effect of RS1 (E72K) mutation on RO development. The BP of GO enrichment analysis revealed that chemical synaptic transmission, regulation of postsynaptic membrane potential, anterior/posterior pattern specification, and nervous system development were downregulated. Moreover, the KEGG pathways associated with patient ROs included gap junction, calcium signaling pathway, GABAergic synapse, glutamatergic synapse, and neuroactive ligand-receptor interaction. Collectively, these findings indicate the presence of synaptic dysfunction in developing ROs with the RS1 (E72K) mutation. This finding is consistent with previous results in mice. Eleftheriou et al. reported that RS1 is associated with the normal formation of glutamatergic synapse [62]. Moreover, the RS1 maintains the integrity of the photoreceptor-bipolar synapse [63, 64]. They showed that the b-wave amplitude of *Rs1*-KO mice was decreased by 50% compared to a 32% reduction in the a-wave at 1 month, suggesting that OPL disruptions, including mislocalization of synaptic structures occurring before measurable loss of synaptic protein, contribute to the b-wave decline at this age. Therefore, we inferred that synaptic dysfunction at the developing retina may explain the abnormal b-wave of EGR. In addition, the Reactome enrichment analysis showed that the expression of Class A/1 (Rhodopsin-like receptors) was downregulated. This further confirms our former findings.

Given that XLRS is influenced by RS1 deficiency, RS1 gene augmentation therapy may confer clinical benefits. Several clinical and preclinical studies on *RS1* gene augmentation therapy in XLRS have been conducted. Presently, two clinical trials have reported shortcomings in BCVA, visual fields, ERG, or improvement in retinal cystic cavities. This may be attributed to insufficient vector penetration, potentially stemming from the use of AAV vitreous injections and the inability to induce adequate expression of the RS1 protein [27, 28]. Although *RS1* gene augmentation therapy in XLRS mice has been reported to decrease retinal schisis cavities effectively, the mouse may be a suboptimal model for XLRS [25, 65]. Here, we used RS1 (E72K) mutation ROs to explore the therapeutic effect of *RS1* gene augmentation therapy in XLRS. AAV2.7m8-RS1 was delivered to RS1 (E72K) mutation ROs at day 70 before the onset of photoreceptor cell maturation. Although the transfection efficiency was less than 20%, the RS1 protein was highly expressed at day 120. Following RS1 overexpression, the NRL expression level was increased in patient ROs at day 90. Moreover, the Rho expression level was upregulated at day 120. This indicated that the *RS1* gene augmentation therapy might rescue the rod photoreceptor developmental delay by regulating NRL in the developmental RS1 (E72K) mutation ROs. Our research indicates that patients with XLRS may experience greater benefits from *RS1* gene augmentation therapy when administered in the early stages of the condition. This underscores the significance of the timing of *RS1* gene augmentation therapy. Boon et al. similarly showed that AAV vector-mediated hCRB2 or hCRB1 gene augmentation partially reinstated the histological phenotype and transcriptomic profile of retinal organoids derived from CRB1 patients [66]. 

The AAV2.7m8 mainly infected the outer layer of ROs, where the photoreceptor most is, even though we did not add a promoter. This may be due to the vector tropisms, which mirrored findings from a previous study [67]. The transduction process of AAV2.7m8 to ROs in the dish mirrors the subretinal delivery method in the animal retina. In our investigation, we illustrated that AAV2.7m8 encounters challenges in fully penetrating the entire RO and reaching its interior. Therefore, we infer that the AAV2.7m8 faces difficulties in reaching photoreceptor cells from the vitreous in the human retina, considering the additional constraint posed by the inner limiting membrane in this process. This result suggests that subretinal injection may be the optimal route for this vector in the human treatment context, although it is relatively invasive when administered intravitreally.

This study has some limitations that should be acknowledged. Using gene editing technology to obtain isogenic control iPSCs for high-quality studies may be more beneficial. Although numerous studies have utilized RO as models to explore disease mechanisms and therapeutic approaches [66, 68–71], there are several aspects that need to be improved in the existing RO models. For example, current ROs lack an RPE monolayer, vascular system, or connective to the brain, which exists in the human retina context. More advanced technology based on hiSPC-derived ROs, such as co-culture and organs-on-chips [72, 73], should be developed to better simulate the human retina. Moreover, the XLRS RO models derived from RS1 mutation patients were analyzed through a combination of single-cell RNA-Seq (scRNA-Seq) and bulk RNA-Seq to explore XLRS pathogenesis. We plan to investigate these in our next research work.

## Conclusions

Overall, our study provides insights into the use of hiPSC reprogramming of patient tissues and hiPSC-derived ROs as models for inherited retinal diseases. This allows for the identification of potential disease mechanisms and therapeutic strategies. Importantly, our results support the idea that RS1 is more than just an adhesion protein maintaining retinal structure. Moreover, it assumes a crucial role in the development of photoreceptor cells and the formation of synapses. In addition, we found that the developmental delay of photoreceptor cells in ROs with the RS1 (E72K) mutation can be rectified through *RS1* gene augmentation therapy. Our investigation adds to our understanding of the role of the RS1 (E72K) mutation in the retina and offers evidence for the timing selection of XLRS therapy.

### Electronic supplementary material

Below is the link to the electronic supplementary material.

Supplementary Material 1

Supplementary Material 2

Supplementary Material 3

Supplementary Material 4

Supplementary Material 5

Supplementary Material 6

Supplementary Material 7

Supplementary Material 8

Supplementary Material 9

## Data Availability

All the data associated with our findings are available upon request to the corresponding author. The data supporting our results are presented in the paper or supplementary materials. The RNA-seq data was published in the Gene Expression Omnibus database (GEO) and the GEO accession number is GSE267405.
