# Supplementary material for: Retinal organoids with X-linked retinoschisis RS1 (E72K) mutation exhibit a photoreceptor developmental delay and are rescued by gene augmentation therapy
Source: Stem Cell Res Ther. 2024 May 31;15:152. doi: 10.1186/s13287-024-03767-4 (PMC11140964; doi:10.1186/s13287-024-03767-4)
Supplement: Supplementary file 1 — Supplementary Material 1 [file 13287_2024_3767_MOESM1_ESM.docx]

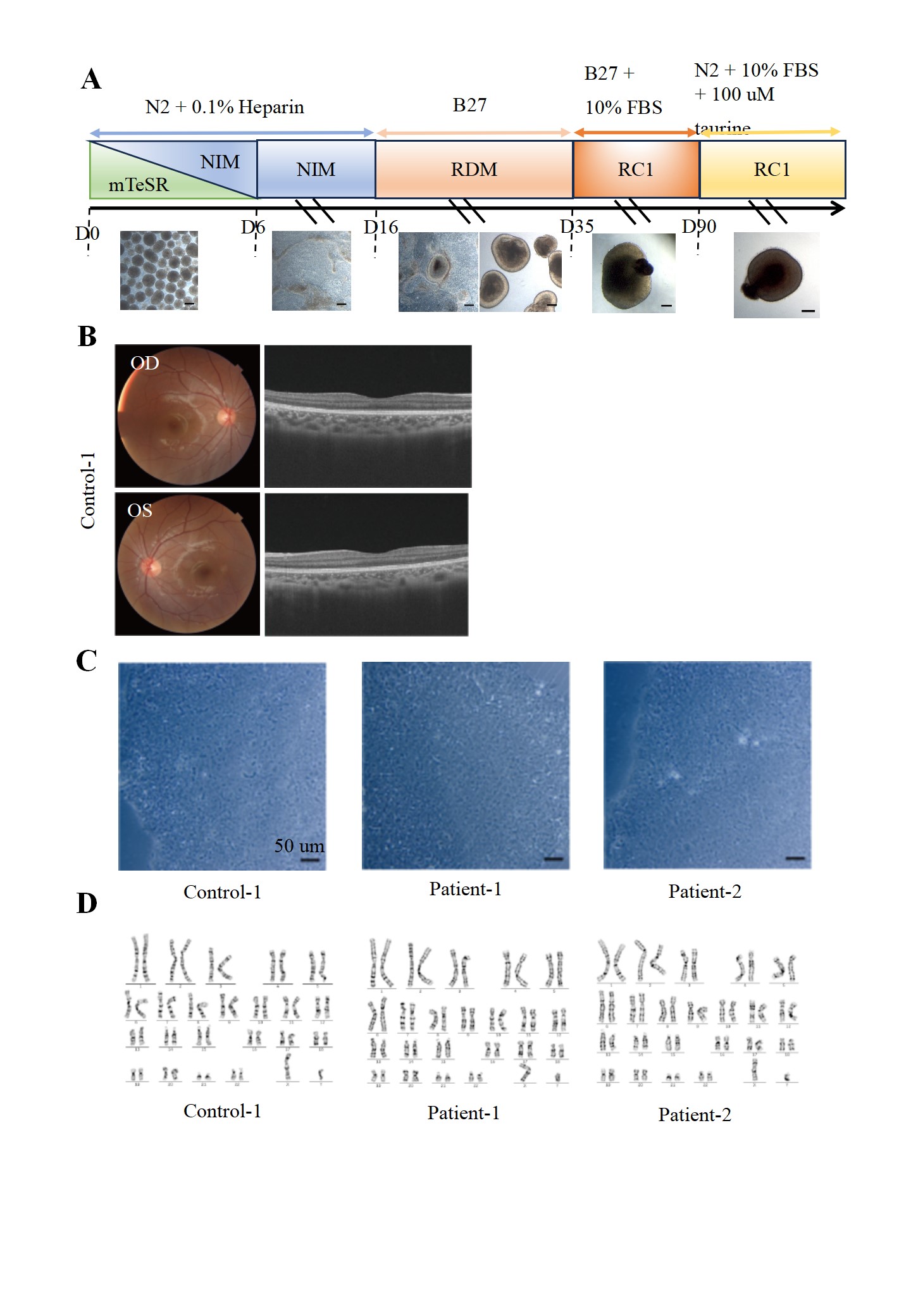


**Figure S1. The workflow of ROs differentiation and characterization of hiPSC lines.** (A). The workflow of ROs differentiation. Scale bar. 200 µm. (B). Fundus photograph and OCT of control-1. (C). Morphology of hiPSCs in control-1, patient-1, and patient-2. Scale bar, 50 µm. (D). Karyotypes of hiPSC lines in control-1, patient-1, and patient-2.
