# Supplementary material for: Retinal organoids with X-linked retinoschisis RS1 (E72K) mutation exhibit a photoreceptor developmental delay and are rescued by gene augmentation therapy
Source: Stem Cell Res Ther. 2024 May 31;15:152. doi: 10.1186/s13287-024-03767-4 (PMC11140964; doi:10.1186/s13287-024-03767-4)
Supplement: Supplementary file 2 — Supplementary Material 2 [file 13287_2024_3767_MOESM2_ESM.docx]

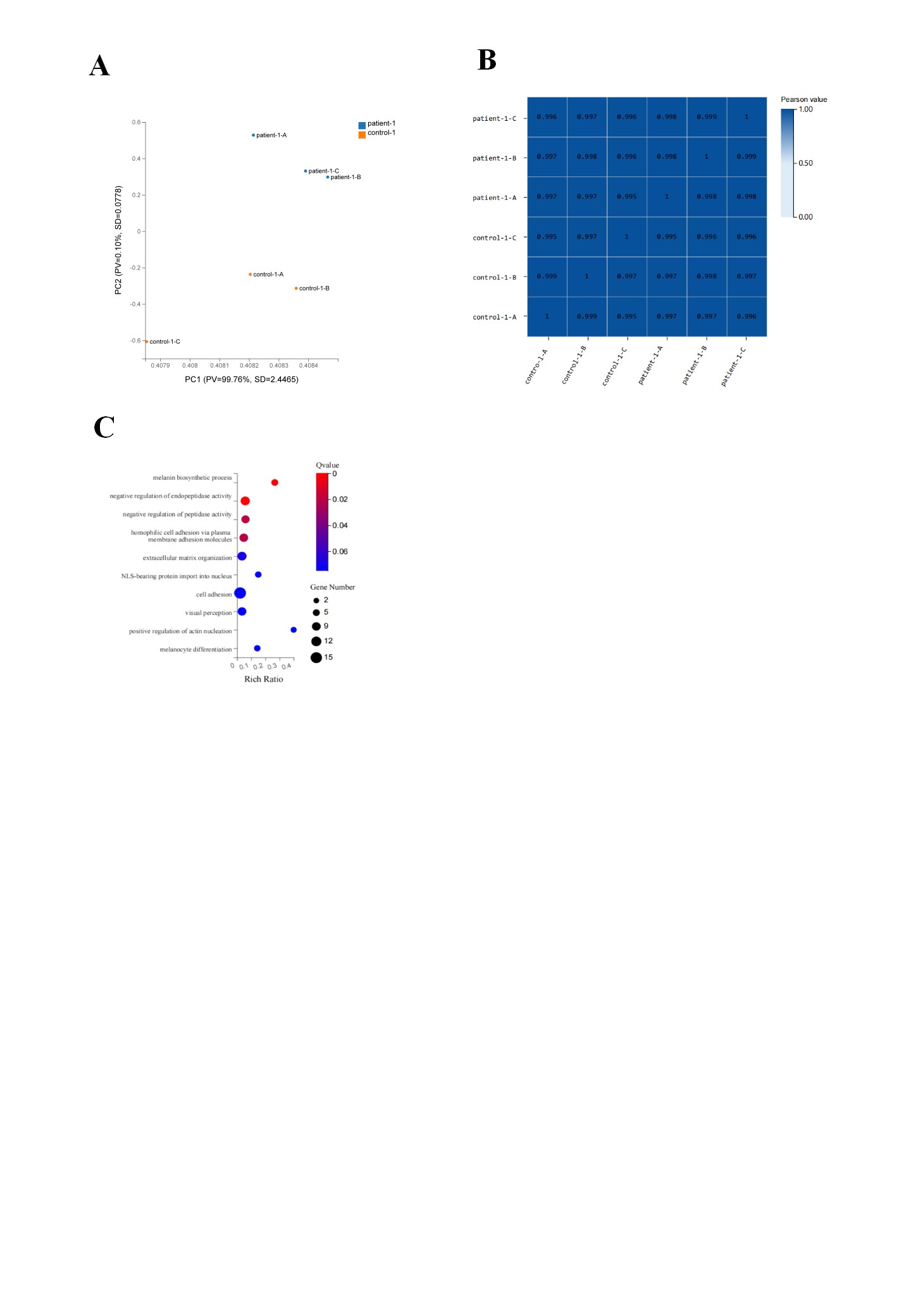


**Figure S2. Characterization of the ROs in the early stage.** (A). Immunofluorescence staining of eye filed transcription factors PAX6, retinal progenitor cells marker CHX10 and OTX2 in ROs at week 6. Scale bar, 20 µm. (B). Full-scale image of ROs with OTX2 staining at week 6. Scale bar, 100 µm. (A, B). The cell nuclei were stained with DAPI (blue).
