# Supplementary material for: Retinal organoids with X-linked retinoschisis RS1 (E72K) mutation exhibit a photoreceptor developmental delay and are rescued by gene augmentation therapy
Source: Stem Cell Res Ther. 2024 May 31;15:152. doi: 10.1186/s13287-024-03767-4 (PMC11140964; doi:10.1186/s13287-024-03767-4)
Supplement: Supplementary file 3 — Supplementary Material 3 [file 13287_2024_3767_MOESM3_ESM.docx]

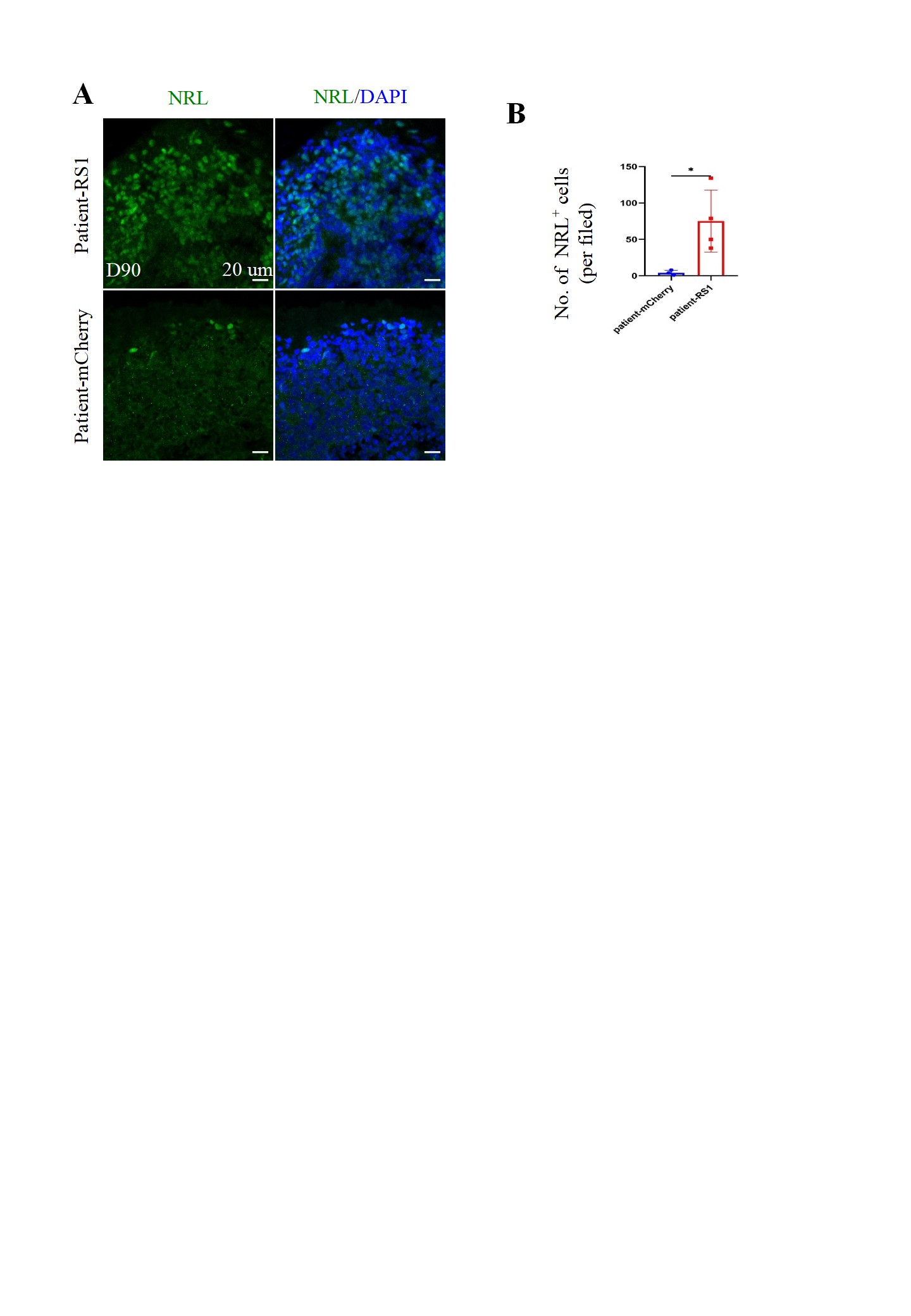


**Figure S3. Identification of the RS1 secretory pathway.** (A). The full-legend blots of RS1 in cells under reducing conditions. (B). The full-legend blots of RS1 in cell culture medium under reducing conditions. (C). The full-legend blots of RS1 in cells under non-reducing conditions. (D). Representative of immunofluorescence staining images of RS1 with ER (GRP94) and Golgi (Golgi97) in HEK293T overexpression cells. The cell nuclei were stained with DAPI (blue). Scale bar, 10 µm.
