# Supplementary material for: Retinal organoids with X-linked retinoschisis RS1 (E72K) mutation exhibit a photoreceptor developmental delay and are rescued by gene augmentation therapy
Source: Stem Cell Res Ther. 2024 May 31;15:152. doi: 10.1186/s13287-024-03767-4 (PMC11140964; doi:10.1186/s13287-024-03767-4)
Supplement: Supplementary file 4 — Supplementary Material 4 [file 13287_2024_3767_MOESM4_ESM.docx]

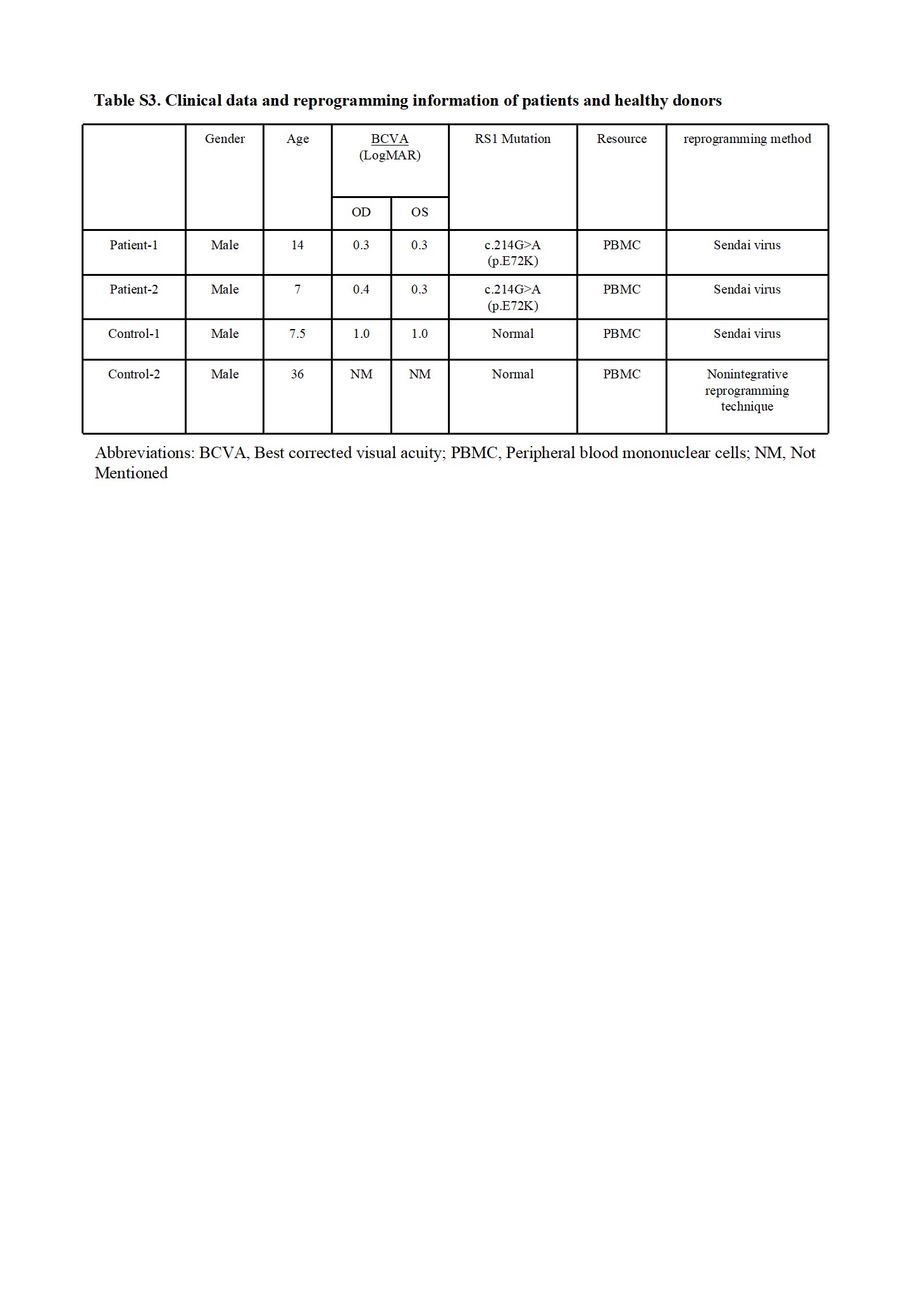


**Figure S4. Characterization of ROs at different stages.** (A). Representative immunofluorescence staining images of Müller glial cells in the control-1 and patient-1 iPSC-derived ROs at day 260 with GS and SOX9 antibodies. Scale bar, 20 µm. (B). Quantification of SOX9 positive cell density in control-1 and patient-1 iPSC-derived ROs at day 260. (C). Quantification of relative fluorescence intensity of GS in the control-1 and patient-1 iPSC-derived ROs at day 260. (D). Representative immunofluorescence staining images of PKCɑ-positive rod bipolar cells and Go(ɑ)-positive ON bipolar cells in control-1 and patient-1 iPSC-derived ROs at day 260. Scale bar, 20 µm. (E). Quantification of PKCɑ and Go(ɑ) positive cell density in the control-1 and patient-1 iPSC-derived ROs at day 260. (F). Full-scale image of ROs with Rho staining at day 260. Scale bar, 100 µm. (G). Staining images of ROs with Caspase-3 at day 120. Scale bar, 20 µm. (H). Quantification of relative fluorescence intensity of Caspase-3 in the control-1 and patient-1 iPSC-derived ROs at day 120. (I). Staining images of ROs with NRL at day 90. Scale bar, 20 µm. (J). Quantification of NRL positive cell density in ROs at day 90. (A, D, F, G, I). The cell nuclei were stained with DAPI (blue).
