# Supplementary material for: Retinal organoids with X-linked retinoschisis RS1 (E72K) mutation exhibit a photoreceptor developmental delay and are rescued by gene augmentation therapy
Source: Stem Cell Res Ther. 2024 May 31;15:152. doi: 10.1186/s13287-024-03767-4 (PMC11140964; doi:10.1186/s13287-024-03767-4)
Supplement: Supplementary file 5 — Supplementary Material 5 [file 13287_2024_3767_MOESM5_ESM.docx]

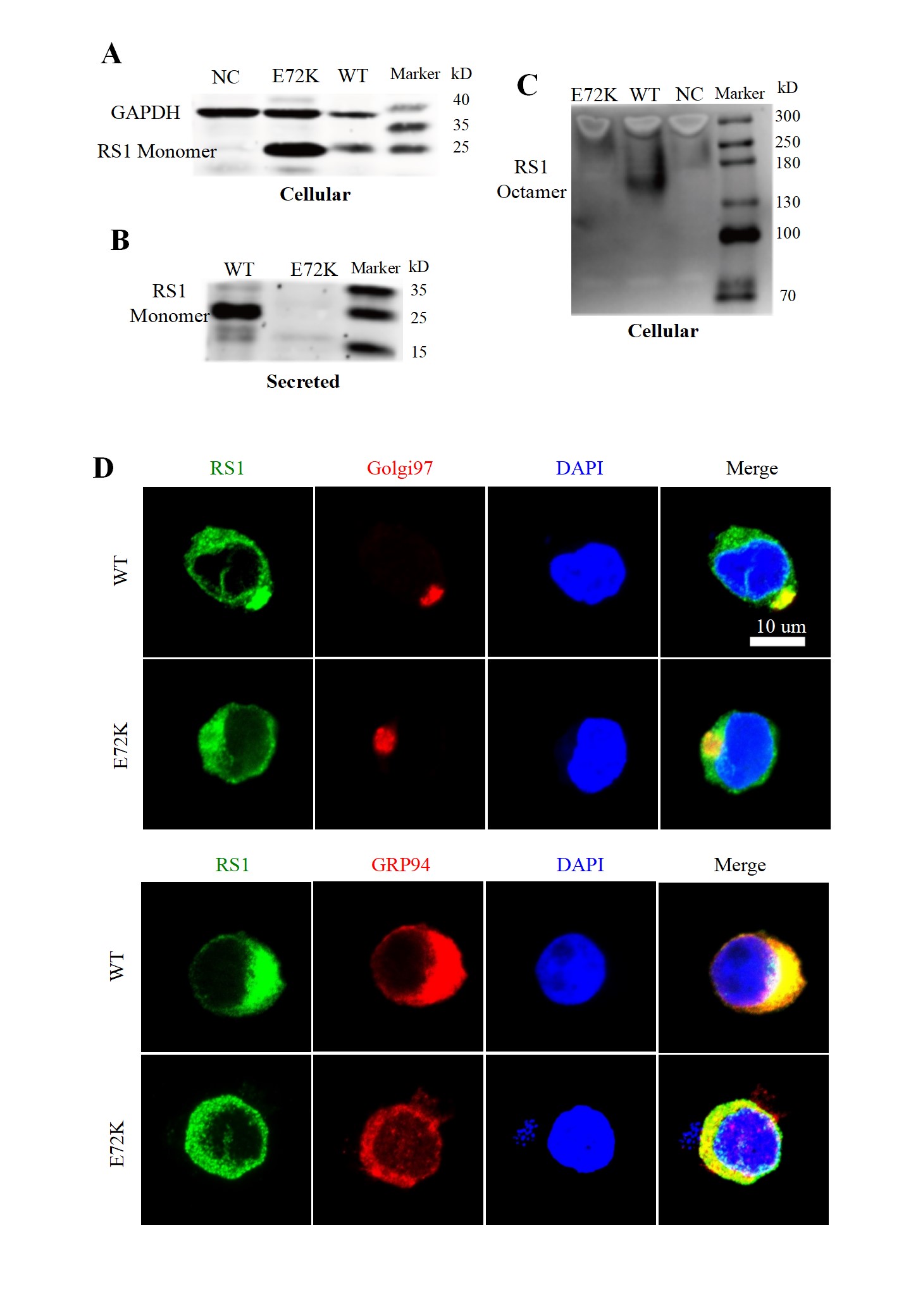


**Figure S5. RNA-seq results.** (A). PCA plot of RNA-seq. (B). Pearson correlation coefficient heatmap of RNA-seq results. (C) The bubble plot of the top 10 GO BPs of upregulated genes.
