# Supplementary material for: Retinal organoids with X-linked retinoschisis RS1 (E72K) mutation exhibit a photoreceptor developmental delay and are rescued by gene augmentation therapy
Source: Stem Cell Res Ther. 2024 May 31;15:152. doi: 10.1186/s13287-024-03767-4 (PMC11140964; doi:10.1186/s13287-024-03767-4)
Supplement: Supplementary file 6 — Supplementary Material 6 [file 13287_2024_3767_MOESM6_ESM.docx]

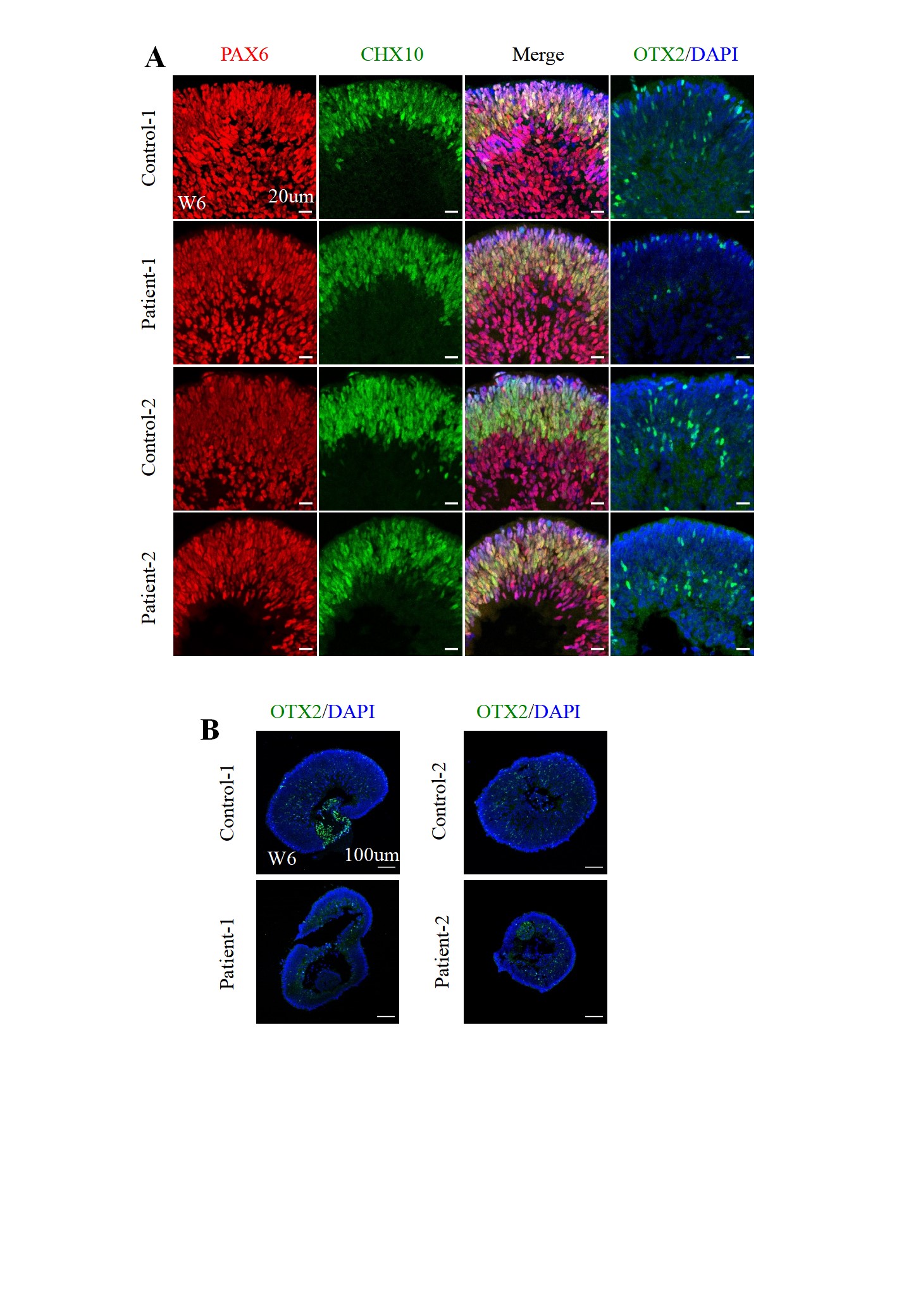


**Figure S6. The expression level of NRL in AAV2.7m8 treated ROs.** (A). Representative immunofluorescence staining images of NRL in patient-RS1 and patient-mCherry ROs at day 90. The cell nuclei were stained with DAPI (blue). Scale bar, 20 µm. (B). The NRL-positive cells were quantified in patient-RS1 and patient-mCheery ROs at day 90.
