# Supplementary figures and images for: Retinal organoids with X-linked retinoschisis RS1 (E72K) mutation exhibit a photoreceptor developmental delay and are rescued by gene augmentation therapy
Source: Stem Cell Res Ther. 2024 May 31;15:152. doi: 10.1186/s13287-024-03767-4 (PMC11140964; doi:10.1186/s13287-024-03767-4)

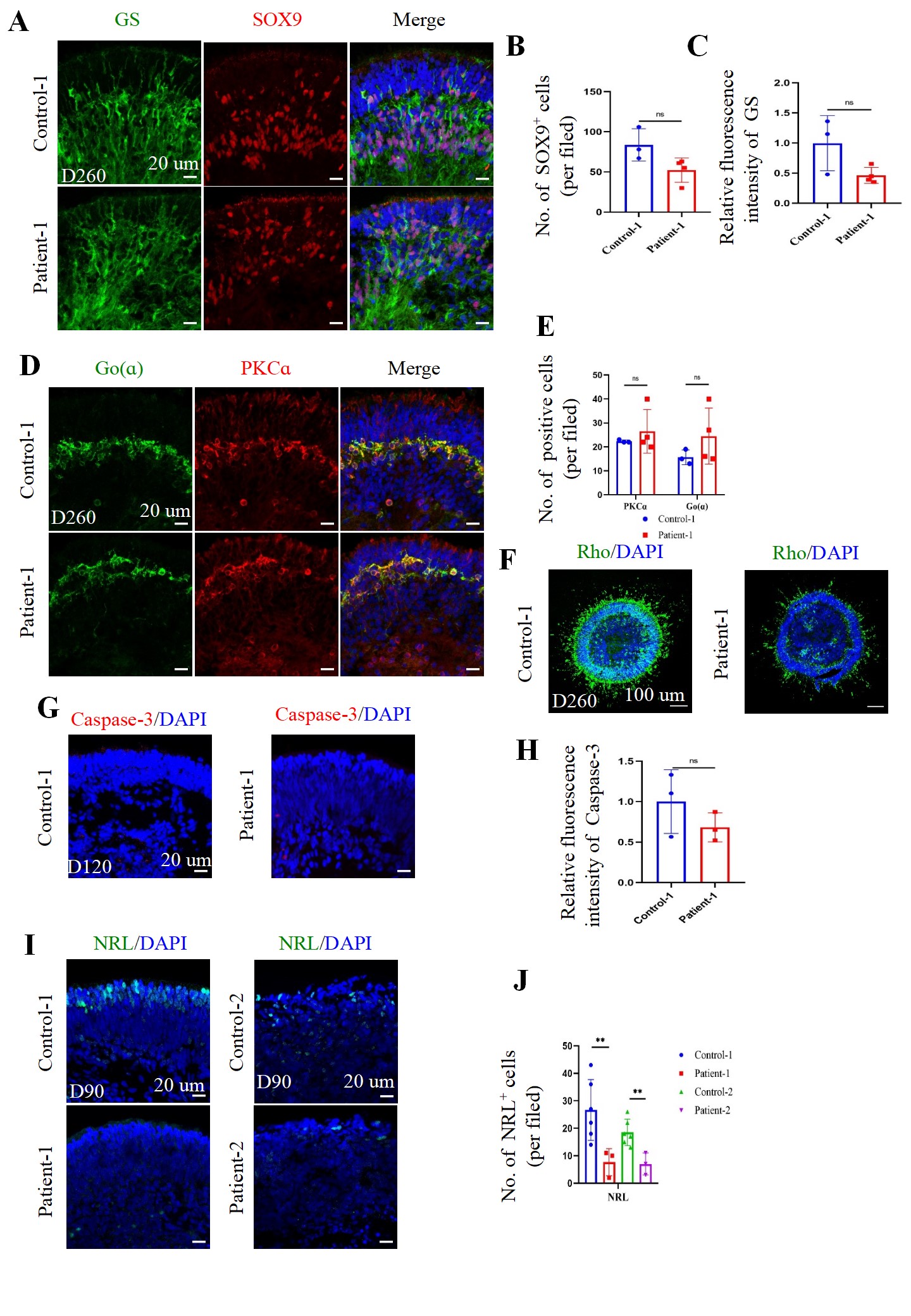

Supplement: Supplementary file 7 — Supplementary Material 7 [file 13287_2024_3767_MOESM7_ESM.docx]
